# Alterations of Ion Homeostasis in Cancer Metastasis: Implications for Treatment

**DOI:** 10.3389/fonc.2021.765329

**Published:** 2021-12-20

**Authors:** Gulimirerouzi Fnu, Georg F. Weber

**Affiliations:** College of Pharmacy, University of Cincinnati Academic Health Center, Cincinnati, OH, United States

**Keywords:** metastasis, ion homeostasis, membrane potential, calcium, potassium channel, sodium channel, chloride, NKCC

## Abstract

We have previously reported that metastases from all malignancies are characterized by a core program of gene expression that suppresses extracellular matrix interactions, induces vascularization/tissue remodeling, activates the oxidative metabolism, and alters ion homeostasis. Among these features, the least elucidated component is ion homeostasis. Here we review the literature with the goal to infer a better mechanistic understanding of the progression-associated ionic alterations and identify the most promising drugs for treatment. Cancer metastasis is accompanied by skewing in calcium, zinc, copper, potassium, sodium and chloride homeostasis. Membrane potential changes and water uptake through Aquaporins may also play roles. Drug candidates to reverse these alterations are at various stages of testing, with some having entered clinical trials. Challenges to their utilization comprise differences among tumor types and the involvement of multiple ions in each case. Further, adverse effects may become a concern, as channel blockers, chelators, or supplemented ions will affect healthy and transformed cells alike.

## Introduction

Genetic changes underlie the transformation process from healthy cells to invasive cancer. The mutator phenotype (1) assures that this phenomenon is sustained throughout the evolution of a tumor. Distinct models have been put forth to explain the evolvement. The big bang model suggests that evolutionary rates in tumors can be variable with periods of punctuated mutational bursts and relative stasis. It proposes that most mutations occur late in tumor development (2). The branched evolution model notes mutations occurring throughout tumor development and selective sweeps to drive clonal development (3). Notwithstanding the plasticity that is generated by the ongoing genetic changes, and which in metastasis may impact angiogenesis, metabolism, immune characteristics, and other factors, there are consistent phenotypic requirements associated with tumor progression. Those constitute potential targets for medical treatment.

While the early steps in cancerous transformation are caused by very specific genetic alterations, namely loss-of-function mutations in tumor suppressor genes or gain-of-function mutations in proto-oncogenes, disease progression is associated with considerable but less-well understood deviations from homeostasis. The genetic basis of cancer progression is not rooted in mutations, but in aberrant expression and splicing of stress response genes (4). Despite variation from tumor heterogeneity, genetic instability/clonal evolution and host factors, consistent genetic adjustments are required within the tumor cells to complete the process of metastasis (5, 6). A major advance in our understanding has been the recognition of entire gene expression programs that are associated with metastases. They are characterized by unique patterns, which distinguish them from the originating tumor as well as from the target host tissue (5, 6). The core program components entail reduced extracellular matrix interactions, increased oxidative metabolism, vascularization/tissue remodeling, and altered ion homeostasis. Among these components, ion homeostasis is substantially skewed in transformed cells as well as in clinical cancer specimens (5, 7, 8). However, the underlying changes in the management of the ionic balance are complex (**Supplement Table 1**). This multifaceted nature has thus far prevented the therapeutic potential of ion channel blockers, chelators, or supplementation of down-regulated ions from coming to fruition. It is important to review the literature specifically for reports on the metastasis-associated disturbance of ion homeostasis in order to define promising drug candidates (**Table 1**).

**Table 1:** Drug candidates.

Ion	Drug Candidate	Action	Characterization	Condition or Disease	Status	Phase	Cancer	Status	Phase
calcium	senicapoc (2,2-bis(4-fluorophenyl)-2-phenylacetamide)	channel blocker		Dehydrated Hereditary Stomatocytosis	recruiting				
	2-APB (2-aminoethyl diphenylborinate)	channel blocker							
	amlodipine besylate	channel blocker	selective for L-type calcium channels	Hypertension/Bioequivalence	completed	4	Metastatic Triple Negative Breast Cancer	completed	1,2
	felodipine	channel blocker	selective for L-type calcium channels	Hypertension/Food Drug Interaction	completed	4, 1			
	manidipine dichloride	channel blocker	selective for L-type calcium channels	Cardiovascular Disease/ Hypertension	completed	4, 3			
	cilnidipine	channel blocker	selective for L-type calcium channels	Hypertension/Stroke/Metabolic Syndrome X	completed	4, 3			
	verapamil	channel blocker		Diabetes / High Blood Pressure	completed	4	Brain Cancer/ Malignancies	completed	2
potassium	4-aminopyridine	channel blocker	general K^+^ channel blocker	Multiple Sclerosis/Non-Arthritic Ischemic Optic Neuropathy	completed				
	TEA	channel blocker	general K^+^ channel blocker						
	imipramine	channel blocker	targets voltage-gated channels	Depression, Dysthymic Disorder	completed	4	HER2 Positive Breast Carcinoma	completed	0
	E4031	channel blocker	targets voltage-gated channels						
	dequalinium	channel blocker	targets voltage-gated channels	Bacterial Vaginosis/Vulvovaginal Candidiasis	completed	4, 3			
	amiodarone	channel blocker	targets voltage-gated channels	Atrial Fibrillation	completed	4			
	psora-4	channel blocker	inhibits *Kv1.3* and *Kv1.5*						
	charybdotoxin	channel blocker	inhibits *Kv1.3*						
	margatoxin	channel blocker	inhibits *Kv1.3*						
	iberiotoxin	channel blocker	targets BK channels						
	charybdotoxin	channel blocker	targets BK channels						
	quinine	channel blocker	targets BK channels	Plasmodium Infections, Anemia	completed	4			
	tetrandrine	channel blocker	targets BK channels	COVID-19	enrolling	4			
	tetraethylammonium	channel blocker	targets BK channels	Hyperlipidemia	terminated	2			
	apamin	channel blocker	targets SK channels						
zinc	TPEN	chelator	membrane-permeable, selective						
									
copper	trientine	chelator	anti-angiogenesis	Wilson Disease	completed	4	Fallopian Tube Cancer / Ovarian Neoplasms Malignant / Primary Peritoneal Cancer	completed	1,2
	salicylaldehyde pyrazole hydrazone	chelator	anti-angiogenesis						
	tetrathiomolybdate			Primary Biliary Cholangitis, Wilson Disease	completed	3	Prostate Cancer, Carcinoma, Colorectal/ Non-Small Cell Lung Cancer	completed	2, 1
	penicillamine			Cystine renal calculi	completed	4	Brain and Central Nervous System Tumors	completed	2
	disulfiram			Alcohol Dependency	completed	4	Metastatic Breast Cancer/Metastatic Pancreatic Cancer	completed	2
	clioquinol			Dermatitis, Eczematous	completed	3	Acute Lymphocytic Leukemia / Acute Myeloid Leukemia / Chronic Lymphocytic Leukemia	terminated	1
iron	ciclopirox olamine	chelator		Onychomycosis, Foot Dermatoses	completed	4	Hematologic Malignancy, Acute Lymphocytic Leukemia/ Advanced Solid Tumors	completed	1
	thiosemicarbazones	chelator		Renal Failure, Renal Artery Stenosis	completed	2	Unspecified Adult Solid Tumor, Protocol Specific, Prostate Cancer/Metastatic Well Differentiated Neuroendocrine Neoplasm	completed / recruiting	1
	tachpyridine	chelator							
	deferiprone	chelator		Cardiomyopathy, Iron Overload, Deteriorating Renal Function	completed	4	Colon Cancer, Breast Cancer, Rectal Cancer, Urethral Carcinoma	completed	2
	deferasirox	chelator	suppression of N-Cadherin	Acute Undifferentiated Leukemia/ Iron Overload	completed / terminated	2	Breast Cancer, Leukemia	terminated	2
	desferrioxamine	chelator	restores E-Cadherin localization	Cardiomyopathy / Iron Overload	completed	4	Acute Myeloid Leukemia/Acute Lymphoblastic Leukemia/ Myelodysplastic Syndrome	terminated	
NKCC	chlorotoxin	channel blocker	Cl^-^ channel blocking peptide				Breast Cancer/Non-Small Cell Lung Cancer/Melanoma/ Brain Neoplasm	completed	1,2
	NPPB	channel blocker	targets outwardly rectifying chloride channels	Cardiac Surgery/Low Cardiac Output/ Natriuretic Peptide B	completed				
VGSC	propranolol		decreases neonatal Nav1.5 expression	Post-Traumatic Stress Disorder, Brain Injuries, Traumatic	completed	4	Invasive Epithelial Ovarian Cancer, Primary Peritoneal Carcinoma, Fallopian Tube Cancer, Cervical Cancer, Pediatric Cancer/Breast Cancer	completed / erminated	1,2/2
	ranolazine			Pulmonary Hypertension, Angina	completed	4	Adenocarcinoma of the Prostate, Bone Metastases, Soft Tissue Metastases	completed	
	phenytoin			Acute Kidney Injury / Impaired Renal Function / Kidney Failure	completed	4	Pancreatic Cancer, Locally Advanced Breast Cancer and Large Operable Breast Cancer/ Metastatic Breast Cancer, Metastatic Pancreatic Cancer	active, not recruiting/ recruiting	2/2,3
	carbamazepine			Bipolar Disorder (BD), Epilepsy, Erythromelalgia	completed	4	Brain and Central Nervous System Tumors, Glioblastoma	completed	1,2
	valproate			Acute Kidney Injury/ Impaired Renal Function /Kidney Failure	completed	4	Advanced Cancer/Prostate Cancer, Breast Cancer, Pancreatic Cancer	completed/ terminated	1, 2
	lamotrigine			Bipolar Disorder (BD)	completed	4	Brain and Central Nervous System Tumors/Malignant Glioma	terminated/ recruiting	2, 4
	ranolazine			Adenocarcinoma of the Prostate, Bone Metastases, Soft Tissue Metastases	completed				
	resveratrol			Allergic Rhinitis (AR), Depression	completed	4	Colon Cancer, Stage I, II, Stage III Rectal Cancer, Stage II, III	completed	1
	ropivacaine			Anesthesia, Conduction / Arthroplasty, Replacement / Postoperative Pain	completed	4	Malignant Neoplasm of Breast	completed	3
	lidocaine			Acute and Chronic-radicular LBP	completed	4	Lung Cancers, Unspecified Adult Solid Tumor, Prostate Cancer	completed	1,2
	mexiletine			Cryptogenic Sensory Polyneuropathy, Ventricular Tachycardia	completed	4			
	flunarizine		vasodilator	Chronic Migraine / Fibromyalgia	completed	4			
	riluzole			Amyotrophic Lateral Sclerosis, Fatigue / Inflammation, Major Depressive Disorder	completed	4	Breast Cancer/ Metastatic Cancer	Withdrawn /terminated	1
	S0154	channel blocker	inhibits expression of *Nav1.6* and *Nav1.7*						
	S0161	channel blocker	inhibits expression of *Nav1.6* and *Nav1.7*						
	naringenin	channel blocker	targets *SCN9A*	Hepatitis C Virus, HCV Infection, Chronic HCV, Hepatitis C	completed	1			
	bumetanide			Heart Failure, Autism	completed	4			
H_2_O		channel blocker	Aquaporin						

The potential uses against metastasis are described in the references in the main text, information on drug trial was retrieved from https://clinicaltrials.gov/ and information on approved drugs comes from https://www.accessdata.fda.gov/scripts/cder/daf/.

Shown are drugs that are under consideration or in clinical trials as anti-metastasis agents, which may counteract the ionic imbalance in disseminating or disseminated cancer cells.

## Ion Gradients

Dysequilibrium is essential for all life. The membranes surrounding cells and cellular compartments play critical roles. Across the plasma membrane, many ions are asymmetrically distributed. The imbalance produces an electrochemical gradient, which is upheld through an energy-requiring, ATP-dependent transport. This electrochemical gradient generates a membrane potential that is utilized to transduce signals and support the transport of multiple molecules. The extent of the membrane potential is determined by the permeability and concentrations of particular ions between the intracellular and extracellular milieus. It changes in response to environmental cues. The proteins that shuttle ions across cell membranes belong to the classes of ion channels, which allow movements down concentration or electrical gradients, and ion pumps, which energy-dependently move ions against those gradients.

- Ion channels enable selective ionic permeability, and their opening prompts a flow of ions down their specific electrochemical gradient (9).

- Transporters can move ions up a gradient. Their activities are energy-requiring.

Much has been written about the altered ion homeostasis in various cancers. In this regard, many reports have focused on the changes in primary tumors or have not distinguished between primary and metastatic growths. In recent years, substantial evidence has amassed showing that the signal transduction and metabolism of disseminated cancer cells are generally dramatically different from their primary lesions. This circumstance makes it important to analyze cancer spread separately from the primary tumors. The present review focuses on metastasis-associated alterations in ionic balance within the cancer cells.

## Components of the Metastatic Process

**Figure 1:**
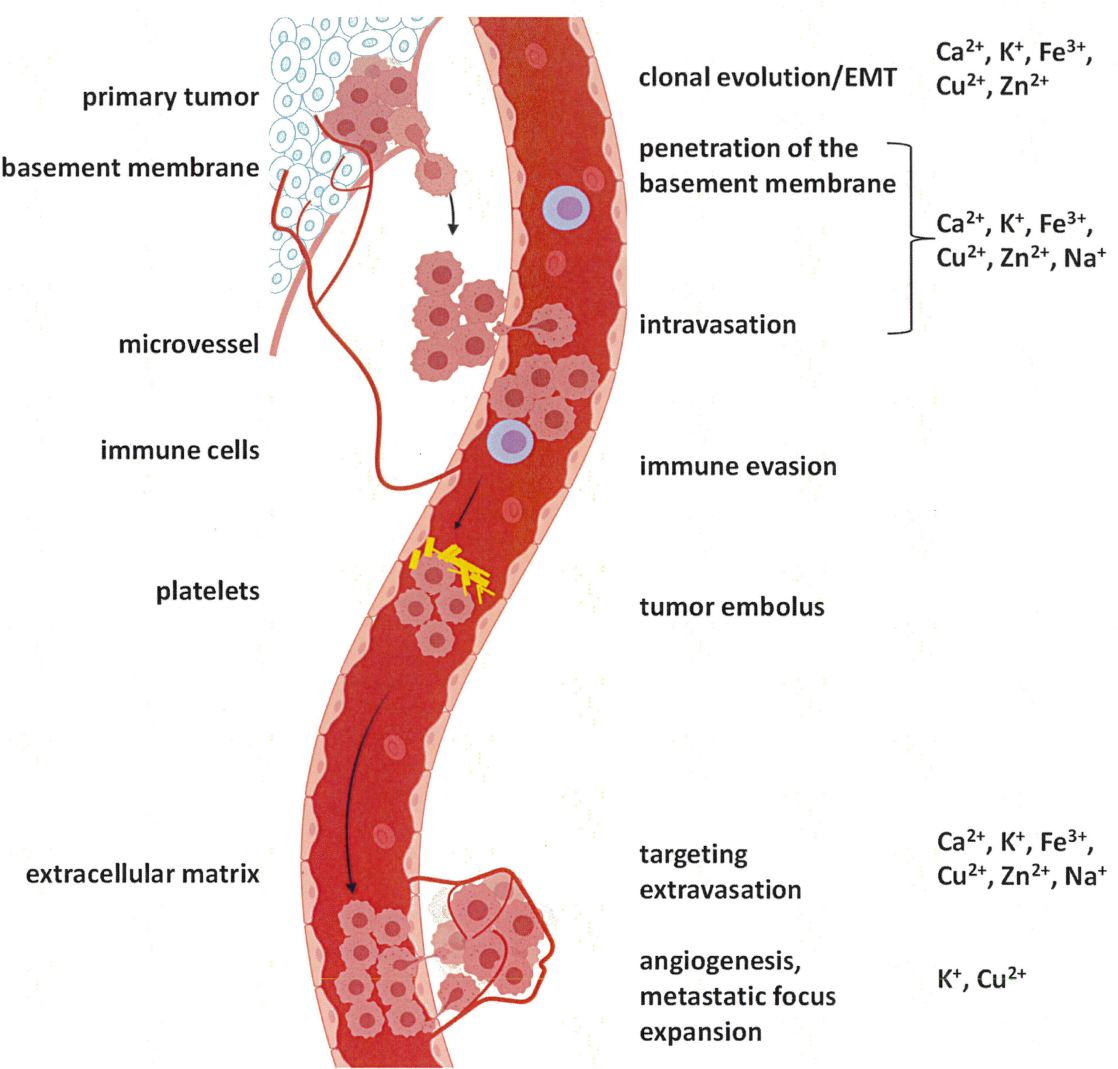
The metastatic cascade. Tumor cells initially break through the basement membrane at the site of origin. From there, they enter the circulation (blood or lymph stream), survive, and enter the tissue at the target site. Preparation of a pre-metastatic niche (10) may be required, but is not depicted (adapted from (11) using BioRender).

The metastatic cascade (**Figure 1**) reflects a process, which is activated secondarily to transformation and is under the control of its own unique gene expression programs (12). The driver genes of this program commonly are activated by aberrant expression or splicing. When mutations are involved, they typically affect the regulatory regions of the relevant genes (13). While cancer spread is supported by a variety of biochemical cascades, certain consistent features are required, including some specific alterations in ion homeostasis. For several components of cancer dissemination, a substantial knowledge base exists in regard to the participating ions. They entail the epithelial-mesenchymal transition, anti-anoikis, and migration/invasion.

Specific ion channels are able to regulate aspects in the induction of the epithelial-mesenchymal transition. Also, changes in plasma membrane ion channel expression arise as a consequence of this process. Prominently involved are maintenance of the sodium ion gradient by Na^+^/K^+^-ATPases and changes in cytosolic free Ca^2+^ (14). Further, transient receptor potential (TRP) channels are essential for the progression of this mechanism (15–17).

The predominant limiting factor to metastatic spread is the death of the tumor cells before their localization in the target organ. Thus, anti-anoikis is a crucial mechanism of cancer dissemination. It involves ionic adjustments. In response to triggers of cell death, the pro-apoptotic proteins *BAK* and *BAX* translocate from the cytosol to the outer mitochondrial membrane, where their oligomerization forms a channel within the membrane, thus effectuating mitochondrial permeabilization and Cytochrome *c* release. In addition to the propensity by the *BAX* proteins to form pores, their interaction with mitochondrial channel proteins, such as the voltage-dependent anion channels, may cause membrane permeabilization. Detached cancer cells can adopt various strategies to compensate for the loss of Integrin signals and counteract anoikis signals (18). Whereas loss of intracellular potassium promotes apoptosis, increases in intracellular calcium may either inhibit or promote apoptosis, depending on the concentration level, location and timing (19). The ionic balance in the disseminating cancer cell impacts its susceptibility to anoikis.

Cell motility is a substantial contributor to metastasis formation. Invading cells are subject to dramatic adjustments in cell shape and volume as they traverse extracellular spaces along stromal tracts and blood vessels. Ion channels facilitate migration by fluxing charged molecules, which prompts the osmotically directed movement of water. Through this mechanism, ion channels can modulate cell volume by effectuating the shrinking or swelling of cellular processes. The influx of ions can protrude the leading edge by triggering osmotic water entry through Aquaporins. At the same time, a release of ions through channels at the lagging edge enables osmotic water release and cell shrinkage. In migrating glioma cells, K^+^ channels and Cl^−^ channels colocalize in caveolar lipid rafts on the invadapodia. The high density of ion channels on the leading edge of migrating cells allows Cl^−^ and K^+^ efflux, and it enables invading cellular processes to shrink and migrate into confined spaces.

Consistent with a spectrum of roles in the response to environmental cues, ion pumps and channel proteins may form macro-molecular complexes with cell adhesion molecules, growth factor receptors, and other signal transduction molecules, thus influencing their functions. Therefore, the ion balance of the cancer cell is intricately linked to diverse other response elements that determine their metastatic success.

## Membrane Potential

General: Membrane potential, reflected in the voltage across the plasma membrane, is maintained through ion transporters or ion channels with high ion selectivity. Directional tumor cell migration is controlled in part by intra- and extra-cellular pH, which determines the ionization state of intra- and extra-cellular proteins, thus affecting their function in physiological or pathophysiological processes.

Functions in Metastasis: Cancer cells possess distinct bioelectrical properties characterized by a more depolarized membrane potential compared to their healthy counterparts (20). Cell migration is an important constituent of the metastatic cascade. It requires a concerted action of ion transporters and channels, cytoskeletal structures, and signaling cascades. Together with Aquaporins, ion transport proteins contribute to tumor cell migration and invasion by bringing about local volume changes and/or by modulating H^+^ signaling (21). Ion channels and transporters control cell volume as well as motility, and the membrane potential has functional roles in cancer cell migration.

Candidate Drug Treatments: Membrane potential may be a clinical prognostic marker for tumors. It can be artificially modified in order to inhibit tumor growth and metastasis (20). Ion transport proteins impress as potential candidate targets for treatment, because as membrane proteins they are easily accessible and are often over-expressed or activated in cancer. A number of drugs are available, which are in clinical use for unrelated conditions and whose anticipated efficacy as anti-tumor agents is under evaluation (21).

## Calcium

General: To develop a strong propensity toward migration, the ubiquitous second messenger Ca^2+^ is an essential mediator. The regulation of cytosolic calcium involves calcium release from intracellular stores in the endoplasmic reticulum or the mitochondria and Ca^2+^ entry from the extracellular space. The modulation of cytoskeletal dynamics is one of the main mechanisms, by which calcium signaling impacts cell motility. Cell migration and invasion are directed by cycles of filopodia stabilization that are followed by maturation into focal adhesions (22). Integrin activation and Integrin inside-out signaling activate L-type calcium channels at the filopodia. These channels support filopodia stability and maturation into Talin-rich adhesions *via* spatially constrained regulation of calcium entry, followed by the activation of the protease Calpain-1 (23).

Voltage-gated calcium channels comprise
- the channels *Ca_V_1.1* through *Ca_V_1.4* entail L-type calcium channels (DHP Receptors), which are high-voltage activated and expressed in the neuro-muscular system
- the members *Ca_V_2.1* through *Ca_V_2.3* entail high-voltage activated P-type/Q-type and N-type calcium channels as well as the intermediate-voltage activated R-type calcium channel; they are mostly present on cells of the central as well as peripheral nervous systems
- *Ca_V_3.1* through *Ca_V_3.3* are T-type calcium channels and low-voltage activated.
Ligand-gated calcium channels comprise
- the inositol trisphosphate (IP_3_) receptors *ITPR1* through *ITPR3*
- the ryanodine receptors, entailing *RYR1* through *3*, that mediate calcium-induced calcium release mostly in myocytes
- the two-pore channels *TPCN1* and *TPCN2*, that mediate NAADP-activated calcium transport across endosomal or lysosomal membranes
- the PKD2 cation channel of sperm that is a non-selective calcium-activated cation channel
- the store-operated channels *ORAI1* through *ORAI3* that are located in the plasma membrane and provide calcium signaling to the cytosol.

**Figure 2:**
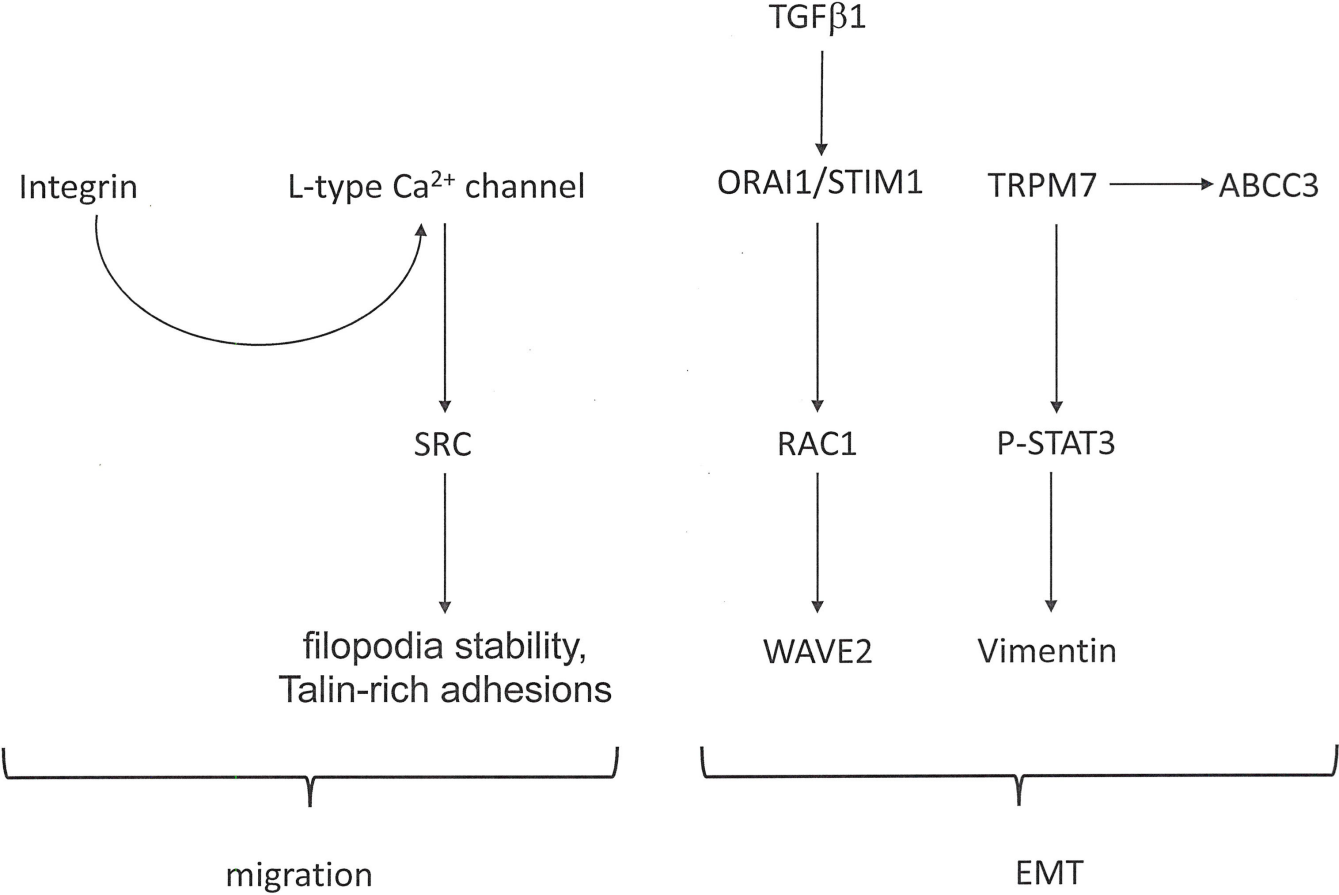
Calcium signaling in cancer metastasis. In cancer progression, calcium flux is involved in facilitating cell migration as well as epithelial-mesenchymal transition (EMT). The Figure shows pathways that are activated in these processes.

Functions in Metastasis: The multi-step process of epithelial-mesenchymal transition (EMT) evolves during the progression of carcinomas and is required for their metastasis formation. The occurrence is accompanied by calcium influx into the cancer cells (**Figure 2**). Stimuli that induce EMT effectuate a transient increase in the cytosolic calcium concentrations of breast cancer cells. Regulators of cellular Ca^2+^ homeostasis, including *ORAI1* (Calcium Release-Activated Calcium Channel Protein 1), *STIM1* (Stromal Interaction Molecule 1), and *TRP* (Transient Receptor Potential) channels, are implicated in tumor cell migration and an aggressive cell phenotype (22). Being involved in store-operated calcium entry, both *ORAI1* and *STIM1*, are essential for breast tumor cell migration and tumor metastasis (24). In colorectal cancer cells, exposure to *TGF-β1* increases the *ORAI1* expression, and *ORAI1* mediates the *TGF-β* induced EMT (25). Migration, invasion, and adhesion ability by osteosarcoma cells depend on *ORAI1* expression. *ORAI1* activates the *RAS*-*RAC1*-*WAVE2* signaling cascade (26). Intracellular calcium is required to mediate the EGF-induced activation of *STAT3*, Vimentin, Twist and N-Cadherin. Plasma membrane channels that contribute to the influx of calcium into cells include transient receptor potential (TRP) ion channels. The expression of *TRPM7* (Transient Receptor Potential-Melastatin-Like 7) regulates the *EGF*-induced *STAT3* phosphorylation and expression of Vimentin (27). In breast cancer, the expression level of *TRPM7* and the formation of metastases correlate positively, suggesting that *TRPM7* contributes to a migratory and invasive phenotype. During epithelial-mesenchymal transition, the transcriptional regulation of ABC transporters (ATP-binding cassette efflux pumps) is altered. *EGF*-induced EMT in breast cancer cells results in the transcriptional up-regulation of the efflux transporter *ABCC3* in a calcium-dependent manner, but independently of *TRPM7* as well as of *ORAI1* and *STIM1* (component of the store-operated calcium entry pathway). Nevertheless, *TRPC1* (Calcium Permeable Ion Channel Transient Receptor Potential Cation Channel, subfamily C, member 1) may contribute to the regulation of basal as well as *EGF*-induced *ABCC3* expression (28).

There is a Ca^2+^ dependence in pro-metastatic behaviors. Cancer cells need to develop an enhanced migratory propensity, for which Ca^2+^ is a crucial regulator. Filopodia play a substantial role in driving the invasiveness of cancer cells. L-type calcium channels are frequently expressed and functional in such cells. At filopodia, these channels are activated by Integrin inside-out signaling and *SRC* activity. L-type calcium channels support filopodia stability and maturation into Talin-rich adhesions *via* spatially constrained modulation of calcium entry, followed by the activation of the protease Calpain-1 (23). These functions lead to cancer cell invasion downstream of integrin signaling.

Driving malignant progression in breast cancer, there are mechano-ionic relationships, which involve mechanically sensitive calcium signaling. Upon loss of cell-cell contacts, there is a rapid initiation of intracellular calcium elevations in exposed cells, followed by a time-dependent increase in Ca^2+^ within cells at progressive distances from the lesional site. Calcium signaling to adjacent cells not located at the edge returns to baseline within seconds. By contrast, calcium elevations at the edge persist for many minutes. Extracellular calcium is required for sustained calcium elevation at the edge, but intercellular signal propagation is dependent on the internal calcium stores. For the latter, extracellular ATP and activation of *P2Y2* receptors are necessary. Aggressively tumorigenic and metastatic cells displays drastic reductions in the calcium signal changes in response to loss of cell-cell contact (29).

Candidate Drug Treatments: Calcium channel blockers are commonly used in clinical practice to treat other diseases. They also are potent inhibitors of filopodia formation by cancer cells (23). Four structurally distinct L-type calcium-channel blockers, amlodipine, felodipine, manidipine, and cilnidipine, substantially suppress the number of *MYO10*-induced filopodia in breast cancer cells, whereas treatment with bumetanide (an inhibitor of the Na^+^/K^+^/2Cl^−^ cotransporter) or zonisamide (an inhibitor of T-type calcium channels, voltage-gated sodium channels and Carbonic Anhydrase) fail to affect filopodia number (23). The efficacy of the channel blocker senicapoc (2,2-bis(4-fluorophenyl)-2-phenylacetamide) as an anti-tumor drug is under evaluation (21). In colorectal cancer cells, blocking the store-operated Ca^2+^ entry with 2-aminoethyl diphenylborinate (2-APB) suppresses the formation of epithelial-mesenchymal transition (25).

## Potassium

General: Potassium channels represent pore-forming transmembrane proteins that enable the flux of K^+^ down an electrochemical gradient. They contribute to the control of membrane potential, cell excitability, cell volume regulation, and apoptosis. These molecules are encoded by 77 genes. They group into 4 classes, comprising voltage-gated potassium channels, calcium-activated potassium channels, inward-rectifier potassium channels and two-pore-domain potassium channels.

- Voltage-gated potassium channels (K_V_) comprise the largest subgroup of potassium channels. Their gating is controlled by changes in membrane potential. This family is further divided into K_V_1-4 channels (Shaker, Shab, Shaw and Shal-like subunits), K_V_7 channels (KCNQ), the silent K_V_5, K_V_6, K_V_8 and K_V_9 subunits (modulators) and K_V_10-12 channels (EAG-like).
- According to their conductance, calcium-activated potassium channels are further divided into big conductance (BK), intermediate conductance (IK), and small conductance (SK) channels.
- Inward rectifying potassium channels (Kir) possess two transmembrane segments flanking one pore loop in each of the four α-subunits.
- Two-pore domain potassium channels (K2P) have two pore domains per α-subunit (two α-subunits form a K2P channel). This type of channel is usually constitutively open as a leak channel for maintaining a negative membrane potential.

Functions in Metastasis: Relevant for metastasis, potassium channels are involved in cell migration and angiogenesis as well as EMT. Expression levels and activities of specific K^+^ channels may increase with tumor grade. Some potassium channels exhibit oncogenic properties and are associated with a malignant cancer phenotype (30).

**Figure 3:**
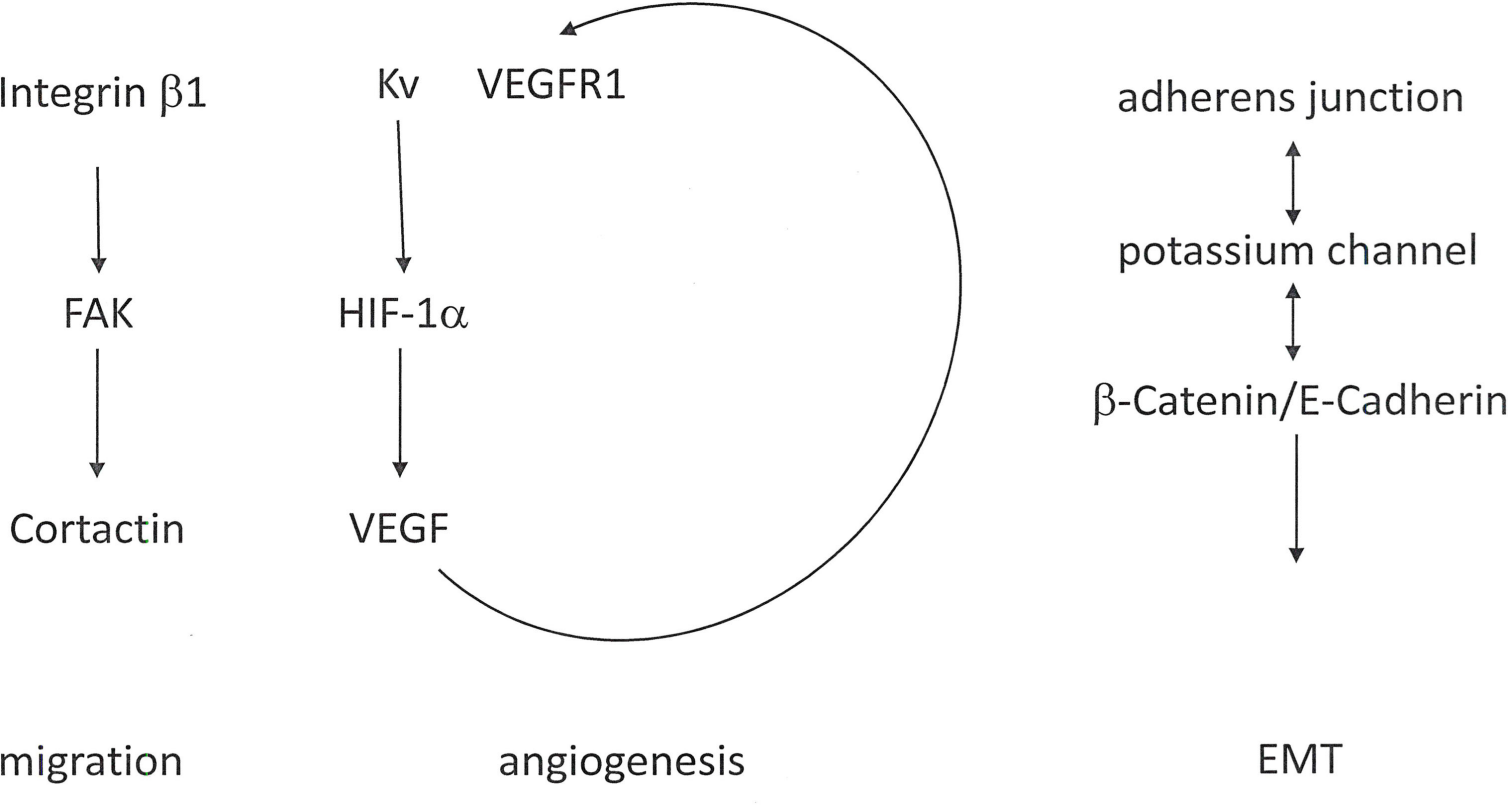
K_V_ channels in cancer metastasis. Alterations in potassium homeostasis contribute to cell migration, angiogenesis and EMT. In the cell membrane, the K_V_ channel associates with Integrin β_1_ and *VEGFR* (Vascular Endothelial Growth Factor Receptor).

- Potassium channels contribute to cell migration, in part through their interactions with β_1_ Integrins (31, 32) and with the Integrin-associated molecules *FAK* and Cortactin (33–36).
- The Integrin/potassium channel complex may be joined by *VEGFR*, thus regulating angiogenesis. Further, potassium channels affect the expression of *HIF-1* and the resulting transcriptional activation of *VEGF* (Vascular Endothelial Growth Factor).
- Another target is β-Catenin signaling (37). An important event characterizing the epithelial-mesenchymal transition is the dislocation of adherens junctions, which causes a loss of cohesion between adjacent cells and consecutively disrupts the epithelial integrity. Potassium channels are directly associated with the β-Catenin/E-Cadherin complex in adherens junctions by several tissues and are required for epithelial integrity. Those potassium channels retain β-Catenin at the cell membrane (**Figure 3**).

The expression of *K_V_1.3* often positively correlates with tumor malignancy (38). However, *K_V_1.3* is also present at the inner mitochondrial membrane, where it may contribute to apoptosis, so that its role in cancer progression is unclear. The contributions by *K_V_1.3* to cancer progression depend on the tumor type. Healthy prostate tissue and benign prostatic hyperplasia display high expression levels of *K_V_1.3*. There is an inverse correlation between *K_V_1.3* and the metastatic ability by prostate cancer cells (39). By contrast, the *K_V_1.3* protein is not abundantly present in healthy breast tissues, whereas most cancers have increased *K_V_1.3* (40, 41), and the levels are high in advanced-stage breast cancer tissues (42). The methylation of the *KCNA3* promoter (which encodes *K_V_1.3*) has been reported to be increased in breast tumors (43). Smooth muscle tumors display a correlation between tumor aggressiveness and the expression of *K_V_1.3*. *K_V_1.3* channels are barely expressed in healthy muscles or indolent leiomyoma but are induced in malignant leiomyosarcoma (44). *K_V_1.5* generates outward delayer rectifying currents with a rapid activation. In leiomyosarcoma as well as rhabdomyosarcoma the expression of *K_V_1.5* is higher and more homogeneous than in healthy muscle tissue (38, 44). The expression of *K_V_1.5* inversely correlates with the grades of gliomas, and this molecule may be a suitable candidate for detection and outcome prediction (41, 45).

*K_V_10.1* expression induces the basal expression levels of *HIF1α* and enhances the response of cells to hypoxia. In cells that express *K_V_10.1*, *HIF1* is activated under only a small decrease in the partial pressure of oxygen. *HIF1* activity causes the transcriptional activation of *VEGF*, which then induces neovascularization. Consistently, tumor cells that express *K_V_10.1* display higher levels of VEGF secretion than controls, and these tumors have increased vascularization (46). Among the voltage-gated channels, *K_V_10.1* is important for the migration of leukemia cells (47) as well as breast cancer cells (48), and its inhibition reduces their migration.

Voltage-gated potassium channels play a prominent but complex role in cancer progression, where the acquisition of cell motility is an indispensable feature of metastasis. When *K_V_11.1* is in its closed state, the channel is associated with Integrin β_1_, in breast cancer resulting in an increase of metastasis. By contrast, when in the open state, the channel association with Integrin β_1_ is reduced, leading to a lessening of breast cancer metastasis (49). In healthy physiology, potassium channels are important for the vascular system and its adaptive mechanisms (50–52). In pathophysiology, potassium channels have a role in tumor angiogenesis. The Vascular Endothelial Growth Factor Receptor 1 (*VEGFR1*) may enter into the macromolecular complex containing β-Integrin and *K_V_11.1* (53). Further, and potentially important for angiogenesis, *K_V_11.1* expression in glioblastoma increases VEGF secretion (54).

When expressed on cancer cells, the potassium channels *EAG* and *hERG*, contribute to cell migration and tumor angiogenesis by controlling VEGF secretion as well as the interactions with Integrin receptors. The K_v_ channel Ether-à-go-go Voltage-Gated K^+^ Channel 1 (*EAG1*), is normally closed when the cells experience resting potential but is open after membrane depolarization. In head and neck squamous cell carcinoma, the expression is elevated at advanced tumor stages (55). In gastrointestinal tumors, increased *EAG1* at the protein level is associated with lymph node metastasis and tumor stage (56, 57). *ERG1* in the colon has increased expression levels in metastatic colorectal cancers. Its abundance on the protein level and its channel activity correlate with the cancer cells’ capacity to invade. The channel mediates the promotion of neovascularization and tumor progression through the interaction with Integrin β_1_ (58, 59). In glioblastoma multiforme, there is channel over-expression in the high-grade tumors. *ERG1* is associated with the promotion of neo-angiogenesis *via* induction of *VEGF* secretion (54).

Calcium-activated potassium channels, including SK, IK, and BK channels, modulate the cell volume, through which they may contribute to cancer cell migration. BK channels (large conductance calcium-activated potassium channels) play a role in cell motility by breast cancer cells, where expression of the *KCNMA1* gene, which constitutes the pore subunit of the BK channel, is up-regulated. Consistently, breast cancer cell migration and invasion is decreased by inhibition of the BK channel (60). Breast cancer and melanoma cells express functional SK channels that promote enhanced migration. The calcium-dependent K^+^ channels *K_Ca_1.1* and *K_Ca_3.1* are important for glioma cell migration (61–63).

Candidate Drug Treatments: The pathophysiology of potassium flux, coupled with the accessibility of potassium channels, the pharmacology of which is well characterized, has pointed to the possibility of targeting potassium channels as a therapeutic modality against cancer metastasis (9). A blocking monoclonal antibody against a potassium channel has shown efficacy (64).

Among the most potent blockers of *K_V_10.1* is imipramine. It has potential as an efficacious agent for the treatment of non-small-cell lung cancer (65). High expression of the gene *KCNH2*, which encodes *K_V_11.1*, is associated with a favorable prognosis in Estrogen Receptor-negative breast cancers. The *K_V_11.1* activator NS1643 can substantially reduce the metastatic spread of triple-negative breast cancers by inhibiting cell motility, counteracting cancer cell stemness, and reprogramming the epithelial-mesenchymal transition through weakening of *WNT*/β-Catenin signaling. The administration of *K_V_11.1* activators may represent a therapeutic modality for the treatment of metastatic Estrogen Receptor-negative breast cancer (37). The *K_V_1.3* protein is increased in breast cancers. Various potassium channel blockers, including dequalinium, amiodarone, and several others inhibit the proliferation of breast tumor cells.

However, there is a caveat to the clinical prospects for modulators of voltage-gated potassium channels. They are potentially compromised because their frequent effects on *K_V_11.1* can lead to acquired long QT syndrome. Without pharmacologic means to distinguish between physiologically and pathologically expressed channels, this adverse effect makes it difficult to design treatments that are directed against, or cross-react with *K_V_11.1*. There is a hopeful prospect in the apparently preferential expression of a unique splice variant of *K_V_11.1* in cancer cells (66, 67). Further, clinical trials have used broad-spectrum potassium channel blockers, such as 4−aminopyridine and its derivatives, which are in use for other diseases. The side effects were rather weak and manageable (9).

Glioma cells over-express calcium-activated potassium channels that contribute to motility. Their tumor grade directly correlates with the BK channel expression levels. The inhibition of large conductance calcium-activated potassium channels (BK) by pharmacologic intervention substantially inhibits glioma cell migration (68). The strongly outwardly rectifying K^+^ current generated by the BK channels is inhibitable by iberiotoxin, charybdotoxin, quinine, tetrandrine, or tetraethylammonium ion (69). Breast cancer and melanoma cells express SK channels with currents that are sensitive to inhibition by 4-aminopyridine (4-AP), apamin or tetraethylammonium (TEA).

The modulator E4031, which blocks potassium channels of the *hERG*-type, may suppress liver metastases from colorectal cancer (59). Inhibition of the channel *EAG* reduces the migration of breast cancer cells as well as acute myeloid leukemia cells, whereas inhibition of *ERG1* reduces the migration of leukemia, melanoma, and thyroid tumor cells (30). Imipramine and astemizole block both *EAG* and *ERG* channels, however with the latter resulting in cardiac risk.

## Iron

General: Iron is an essential element. Efficient electron transfer pays tribute to the importance of iron as a cofactor for enzymes, many of which are involved in DNA reduplication. Therefore, iron often accumulates in tumor tissues. However, its biological activity largely stems from cycling between the ferrous (Fe^2+^) and ferric (Fe^3+^) states, which conveys to iron a complex role in cancer progression, depending on the metabolic context. Redox metabolism and peroxide generation have different importance at various stages of malignancy (70, 71). This may explain the spectrum of observations reported in the literature with regard to the roles of iron in cancer metastasis.

**Figure 4:**
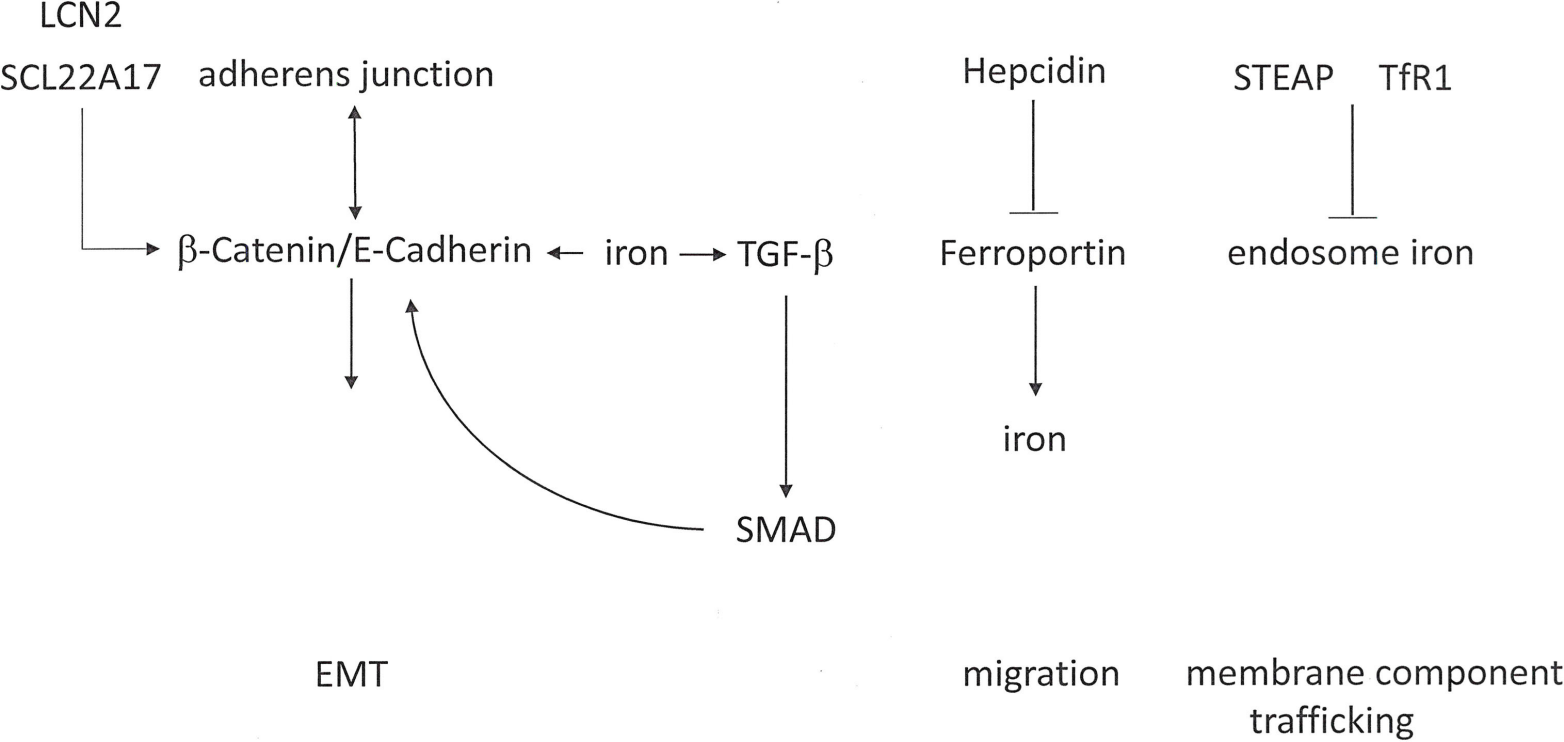
Iron signaling in cancer metastasis. Iron plays complex roles in epithelial-mesenchymal transition (EMT), cell migration, and membrane component trafficking.

Functions in Metastasis: Epithelial-mesenchymal transition may be stimulated by iron loading and can be reversed by iron deprivation (**Figure 4**). In lung cancer cells, iron loading with ferrous sulfate (FeSO_4_) induces migration and invasion (72). The exposure of colon cancer cells to ferric chloride causes them to convert to a mesenchymal phenotype with down-modulated E-Cadherin-mediated cell-cell junctions and up-regulated invasiveness. By contrast, iron chelation restores E-Cadherin localization, causing the cells to become more compact and epithelial-like while also displaying substantially reduced invasion (73). Iron supplementation induces the expression of *TGF-β* and its receptors, thus promoting *SMAD* transcriptional activity, in conjunction with stabilization of β-Catenin to support of its accumulation (74, 75). Excess iron may induce the expression of Matrix Metalloproteinases, which are required for degradation of the extracellular matrix during cancer cell invasion (76). The generation of reactive oxygen species through Fenton reactions can stimulate cell migration, angiogenesis, and cancer stem cell-aggressiveness.

On the basis of abnormal expression in some cancers, the iron-binding protein Lipocalin-2 (*LCN2*) may play a substantial role in metastasis. Within the cerebrospinal fluid-filled leptomeninges, transformed cells encounter non-trivial micro-environmental challenges, including sparse micronutrients and inflammation. Cancer cells, but not macrophages, within the cerebrospinal fluid express Lipocalin-2 and its receptor *SCL22A17*. The resident macrophages secrete inflammatory cytokines that induce *LCN2* expression in the cancer cells. Consecutively, cancer cell expansion is supported by the *LCN2*/*SLC22A17* system. This mechanism represents a potential target for iron chelation therapy (77). Lipocalin-2 over-expression in breast cancer cells enhances migration, invasion, and metastasis. The underlying mechanism may be the down-regulation of the *PI3K*/*AKT* pathway (78). However, conflicting observations have been reported in *RAS*-transformed breast cancer, where the *RAS*/*MAPK* pathway mediates EMT in part by increasing E-Cadherin phosphorylation and ensuing degradation. In *RAS*-transformed mesenchymal tumor cells, Lipocalin-2 can increase E-Cadherin expression, convert to an epithelial phenotype, and suppress cell invasiveness and metastases (79).

The iron export pump Ferroportin constitutes the sole mechanism for the export of non-heme iron from vertebrate cells. The molecule is expressed mostly on macrophages and enterocytes, where it facilitates the delivery of iron to the plasma. The 25-amino acid peptide hormone Hepcidin acts as a master regulator of systemic iron homeostasis. In situations that lead to an excess of iron, Hepcidin binds to Ferroportin and triggers its degradation, which prevents intestinal iron absorption and restricts the release of iron from macrophages. In prostate cancer cells, iron levels are elevated due to Hepcidin-dependent Ferroportin degradation. Such cells are characterized by elevated migratory capacity compared to cells deficient in Hepcidin (80). In comparison to healthy mammary epithelial cells, the Ferroportin protein levels in malignant breast cells are decreased. This is due to a reduction in Ferroportin mRNA and an increase in Hepcidin. The lower Ferroportin gene expression in breast cancers is associated with a substantial shortening in metastasis-free and disease-specific survival, independently of other breast cancer risk factors (81).

Endosomal trafficking contributes to metastasis by recycling components of the plasma membrane (following their internalization *via* Clathrin-dependent/independent vesicles), which is important to establish the polarity of cells and promote cell migration. A link between iron and cancer progression relates to the STEAP family of Metalloreductases, which includes *STEAP1*, *STEAP2*, *STEAP3* and *STEAP4*. Among them, *STEAP2* and *STEAP3* are abundantly expressed in prostate tumors. STEAP family members co-localize with the Transferrin Receptor 1 (*TfR1*), a mediator of Transferrin-mediated iron uptake, which is up-regulated in many cancers. *TfR1* proteins may lower the ferric iron delivered into the endosome *via* Transferrin and conversion to ferrous iron. This mechanism supports the ensuing exit of iron from the endosome into the cytosol *via DMT1*. The up-regulation of *STEAP2* and *STEAP3* expression in cancers may foster the transformed phenotype through their ability to promote iron assimilation (81).

The heme-degradation enzyme Heme Oxygenase-1 exerts antioxidant and immune-modulatory functions. Its role in cancer progression is unclear.

- In breast cancer cells, the activity of Heme Oxygenase-1 (*HO1*) increases the intracellular iron levels, thus inducing migration and invasion of breast cancer cells (82). Conversely, Heme Oxygenase-1 may inhibit lung metastases in a murine breast cancer model. Associated indices for epithelial-mesenchymal transition are inhibited. The *HO-1*/*Notch1*/*Slug* pathway mediates an anti-metastatic effect (83).
- Within the host, Heme Oxygenase-1 in myeloid cells facilitates the formation of the pre-metastatic niche. This impacts the magnitude of tumor cell extravasation and colonization at the metastatic target sites during the early phase of metastasis (84). However, conflicting observations suggest that Heme Oxygenase-1 expression in the host can inhibit melanoma lung metastases (85).

Ferroptosis (86) is an iron-dependent form of programmed cell death, which is reliant on a combination of lipid, amino acid, and iron metabolism. The defining characteristics of this form of cell death entail the presence of redox-active iron, oxidizable phospholipids acylated with polyunsaturated fatty acids, and defective lipid peroxide repair processes (87). The cellular iron levels are determinants for the sensitivity of cells to ferroptosis.

Candidate Drug Treatments: Iron depletion can reverse transformed cells to an epithelial-like state, which diminishes epithelial-mesenchymal transition-dependent migration, invasion and metastasis (88–90). Treatment of colon cancer cells with the iron chelator desferrioxamine restores E-Cadherin localization. The cell morphology becomes more compact and epithelial-like, which is accompanied by a reduced potential to invade (73). Exposure of esophageal cancer cells to the iron chelator deferasirox inhibits migration and invasion through the suppression of N-Cadherin (91). Therefore, iron chelation has been considered a viable treatment strategy to counteract the invasion by cancer cells (76). Various agents are under study. They include desferrioxamine, deferasirox, deferiprone, tachpyridine, thiosemicarbazones, and ciclopirox olamine. However, the results with these drugs have been mixed (76).

## Copper

General: Copper is an essential micronutrient, which is absorbed through food and is vital for several biological functions in small quantities. Free copper ions, however, are toxic and thus all copper in the body is bound to proteins.

**Figure 5:**
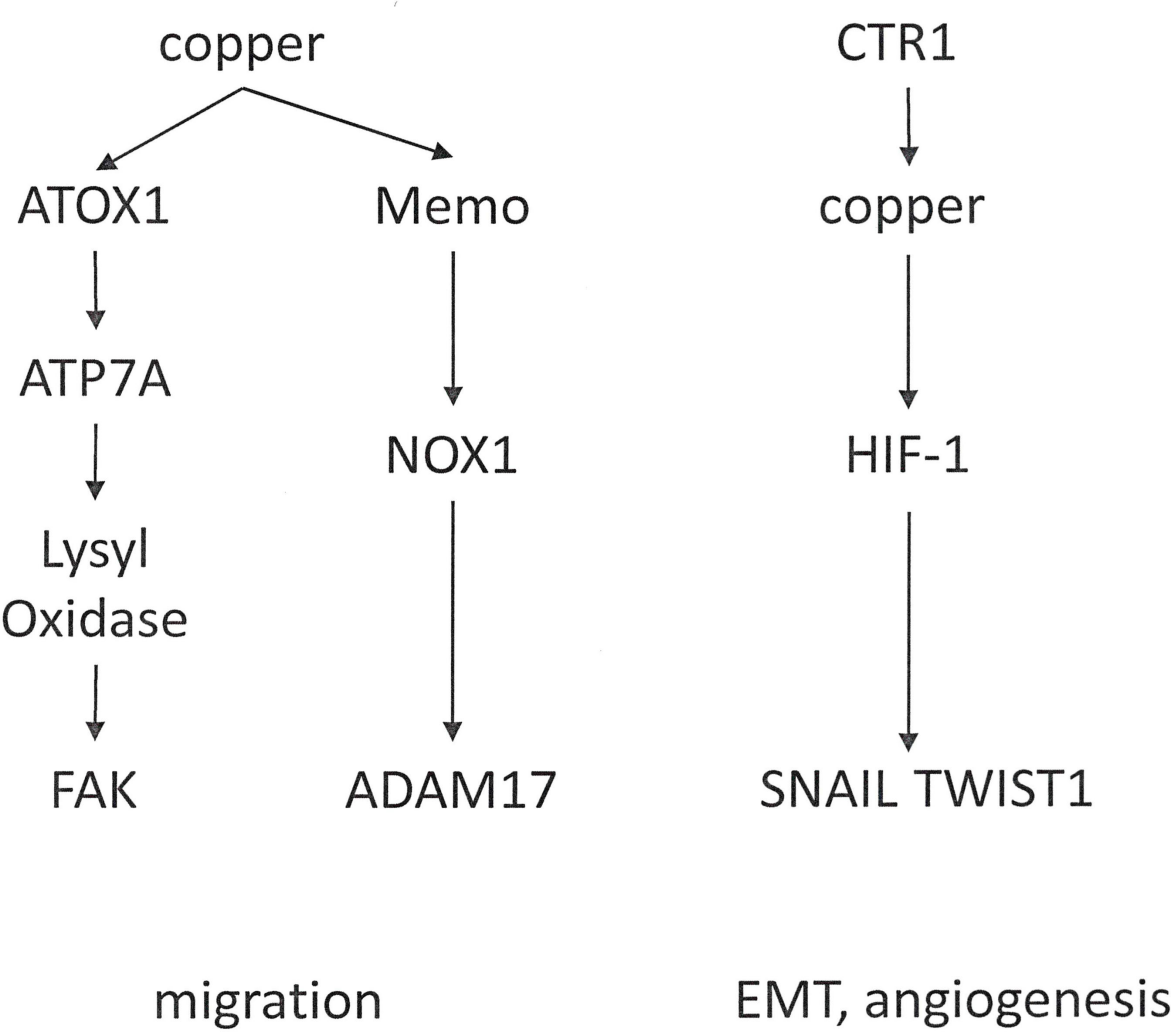
Distinct functions of copper in cancer progression. Copper mediates cell migration. Through a different mechanism, it can effectuate epithelial-mesenchymal transition and angiogenesis.

Functions in Metastasis: Copper is essential to metastatic cancer progression, as it may have a role in EMT (92) and in the formation of the tumor microenvironment. Copper is also important for angiogenesis (93–95) and through this mechanism contributes to the formation of the pre-metastatic niche (**Figure 5**).

In breast cancer cells, *ATOX1* supports the metastatic process. Among the copper-binding proteins, *ATOX1* displays a high concentration in breast cancer cells. It transports copper to other intracellular proteins, which depend on this metal for enzymatic functions. *ATOX1* is localized at the leading edge of moving transformed cells. It promotes cell motility by stimulating a chain reaction of copper delivery involving *ATP7A* (also a copper transport protein) and *LOX* (the enzyme Lysyl Oxidase). *LOX* depends on copper in order to function, and *LOX* is encompassed in extracellular processes that facilitate breast cancer cell movement (96). *LOX* and LOX-Like Oxidase (*LOXL*) are copper-dependent metalloenzymes, which are activated by the copper transporter *ATP7A*. Effectuated downstream is the phosphorylation of Focal Adhesion Kinase (FAK) and myeloid cell recruitment to the lungs in breast and lung cancers. In breast cancer patients, high *ATP7A* expression is a predictor for reduced survival (97). Implying therapeutic potential, the silencing *LOX* reduces the metastatic spread of breast carcinoma cells (98).

The copper-dependent redox enzyme Memo promotes an oxidized intracellular milieu. Further, Memo is important for the sustained production of the reactive oxygen species O_2_^−^ by NADPH Oxidase 1 (*NOX1*) in breast cancer cells. It contributes importantly to the migration and invasion of breast cancer cells as well as spontaneous lung metastasis. Memo abundance is increased in over 40% of primary breast tumors, it correlates with clinical parameters of aggressiveness, and is serves as an independent prognostic factor of early distant metastasis (99). Various endogenous metabolic enzymes generate reactive oxygen species, which play roles in a variety of pathophysiologic processes, including cancer. *NOX1* is highly expressed in the colon tissue and supports colorectal cancer metastasis by impacting the stability of *ADAM17* (100).

Candidate Drug Treatments: Copper depletion has emerged as a potential therapeutic strategy in the treatment of metastatic cancer.

- Selective chelators may work by affecting the bone marrow-derived cells that are critical for the pre-metastatic niche, where hematopoietic precursor cells recruit endothelial precursors. In this process, copper is an important contributor.
- Anti-angiogenesis is also a possible mechanism of action. The chelator trientine may suppress angiogenesis in hepatocellular carcinoma (101). The copper complex of a synthetic salicylaldehyde pyrazole hydrazone derivative induces anti-angiogenesis *via* endothelial cell apoptosis (102).

The copper chelation agents penicillamine, disulfiram, and clioquinol have been tested in various preclinical and clinical studies (103). In a clinical trial, triple-negative breast cancer patients, who were copper-depleted by tetrathiomolybdate, showed a significantly reduced risk of relapse, whereas in advanced kidney cancer only stable disease was achieved with this agent (104, 105).

## Zinc

General: Zinc is a micronutrient required for cell survival and proliferation. It is also a constituent of metalloproteins, and many transcription factors have zinc finger domains. These functions suggest a possible role in malignant transformation. However, there are conflicting reports on the effects of zinc on primary tumors. Similarly, the published research studies are divided regarding the effect of zinc on cancer spread. The apparent inconsistencies in the literature could be a consequence of differences among cancer types, with prostate cancer diverging from other malignancies by reducing the Zinc metabolism.

Functions in Metastasis: Zinc can modulate the organ distribution and viability of metastatic tumor cells (106). Elevated levels of zinc in the host result in increased melanoma survival in the liver, spleen and lungs.

**Figure 6:**
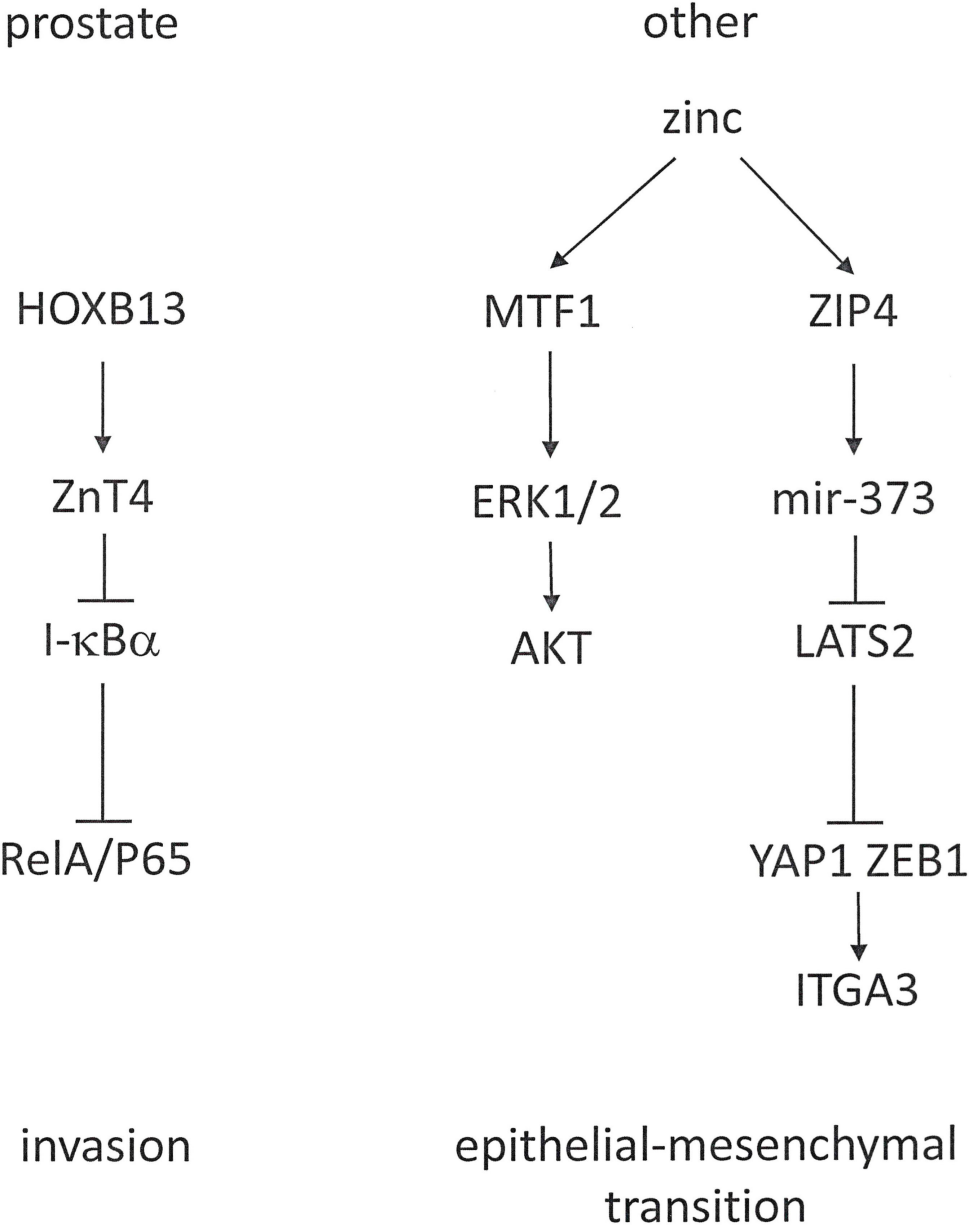
Cancer-type-specific roles for zinc in tumor progression. Zinc metabolism has a different role in prostate cancer, where it is reduced (left), compared to other malignancies, which increase zinc utilization (right).

The transcriptional co-activator *YAP1* is a Hippo pathway effector, which engages in a complex with *ZEB1* to activate the transcription of *ITGA3* (Integrin α3). The zinc transporter *ZIP4* activates *ZEB1* and YAP1 through distinct mechanisms. It activates miR-373 and inhibits the *YAP1* repressor *LATS2* (Large Tumor Suppressor 2 Kinase), in effect up-regulating *YAP1* expression (**Figure 6**). Through this pathway, zinc promotes EMT and metastasis in pancreatic cancer (107). Similarly, zinc is a contributor to ovarian tumor metastasis by promoting EMT through a *MTF1* (Metal Response Transcriptional Factor-1) mediated pathway that involves *ERK1*/*2* and *AKT* (108).

Expression of the zinc transporter *ZIP10* is linked to the metastasis by breast cancer into lymph nodes. The levels of *ZIP10* mRNA are higher in more invasive breast cancer cells. Zinc and *ZIP10* make essential contributions to the migratory activity of highly metastatic breast cancer cells. Therefore, *ZIP10* is a candidate marker for the metastatic phenotype of breast cancer and a potential target for treatment intervention (109). However, it has been unclear whether *ZIP6*/*LIV1* is engaged in the metastasis of breast cancer to the lymph nodes (110, 111).

Prostate cancer cells experience a substantive decrease in intracellular zinc. Whereas the healthy prostate accumulates higher concentrations of this mineral than any other soft tissue (112), the zinc concentrations in prostatic epithelial tumors are markedly reduced and continue to diminish during progression toward androgen-independent growth. *ZIP1* is a suppressor gene for prostate cancer (113). In castration-resistant prostate cancer, *HOXB13* is involved in progression. Among its androgen-independent target genes for up-regulation is the zinc export transporter *ZnT*. *ZnT* proteins belong to the cation diffusion facilitator family. These molecules either export zinc out of cells or sequester zinc in intracellular compartments. *HOXB13* positively targets *ZnT*, but not the influx transporter *ZIP1*. Consequently, it regulates the intracellular abundance of zinc. *HOXB13* stimulates prostate cancer cell invasion, which is inhibitable by the suppression of *ZnT4*. The decrease in intracellular zinc levels resulting from *ZnT* up-regulation reduces *I-κBα* and increases the nuclear translocation of *RelA*/*P65* (114).

Candidate Drug Treatments: Zinc is a contributing factor in ovarian tumor metastasis, where it supports the epithelial-mesenchymal transition. Zinc depletion with the membrane-permeable selective chelator *TPEN* (N,N,N’,N’-tetrakis-(2-pyridylmethyl)-ethylenediamine) may be an approach for ovarian cancer therapy (108).

## Sodium

General: All of the Na^+^ channels and transporters utilize the electrochemical sodium gradient to drive the transport of other ions. Thus, they contribute to maintaining cellular pH.

- Voltage-gated sodium channels (VGSCs) are large protein complexes, which contain a pore-forming α subunit and smaller non-pore-forming β subunits. These channels encompass a multi-gene family of 9 distinct functional members (*Na_V_1.1*- *Na_V_ 1.9*, encoded by genes *SCN1A*-*SCN11A*) coding for the α-subunits, plus 4 auxiliary β-subunits (β1-β4, encoded by genes *SCN1B*-*SCN4B*), of which one or two associate with an α-subunit. Together, they modulate channel expression and activity in the plasma membrane. Voltage-gated Na^+^ channels have two distinct modes of operation, entailing conducting and non-conducting. The conducting mode generates two kinetically different currents that are either transient (INaT) or persistent (INaP). INaP occurs in growing tumors, is promoted by hypoxia, and its activity may increase the levels of Na^+^ in cancer tissues. INaP has been considered a potential anti-metastatic target (115, 116). The α or β subunits of VGSCs may be involved in the non-conducting mode *via* protein-protein interactions as well as immunoglobulin-like cell adhesion effects.
- A group of exchange proteins contributes to the transport of sodium ions. Among them are the Na^+^/H^+^ exchanger (*NHE1*), the Na^+^/HCO_3_^-^ cotransporter, and the Na^+^/K^+^/2Cl^-^ cotransporter (*NKCC*).

Functions in Metastasis: Despite a substantial knowledge base on the regulation of their expression and function in healthy excitable cells, how sodium channels convey pro-metastatic behavior to cancer cells is less well documented (117). Their activity results in potentiation of cellular endocytosis, directional motility, and invasion.

**Figure 7:**
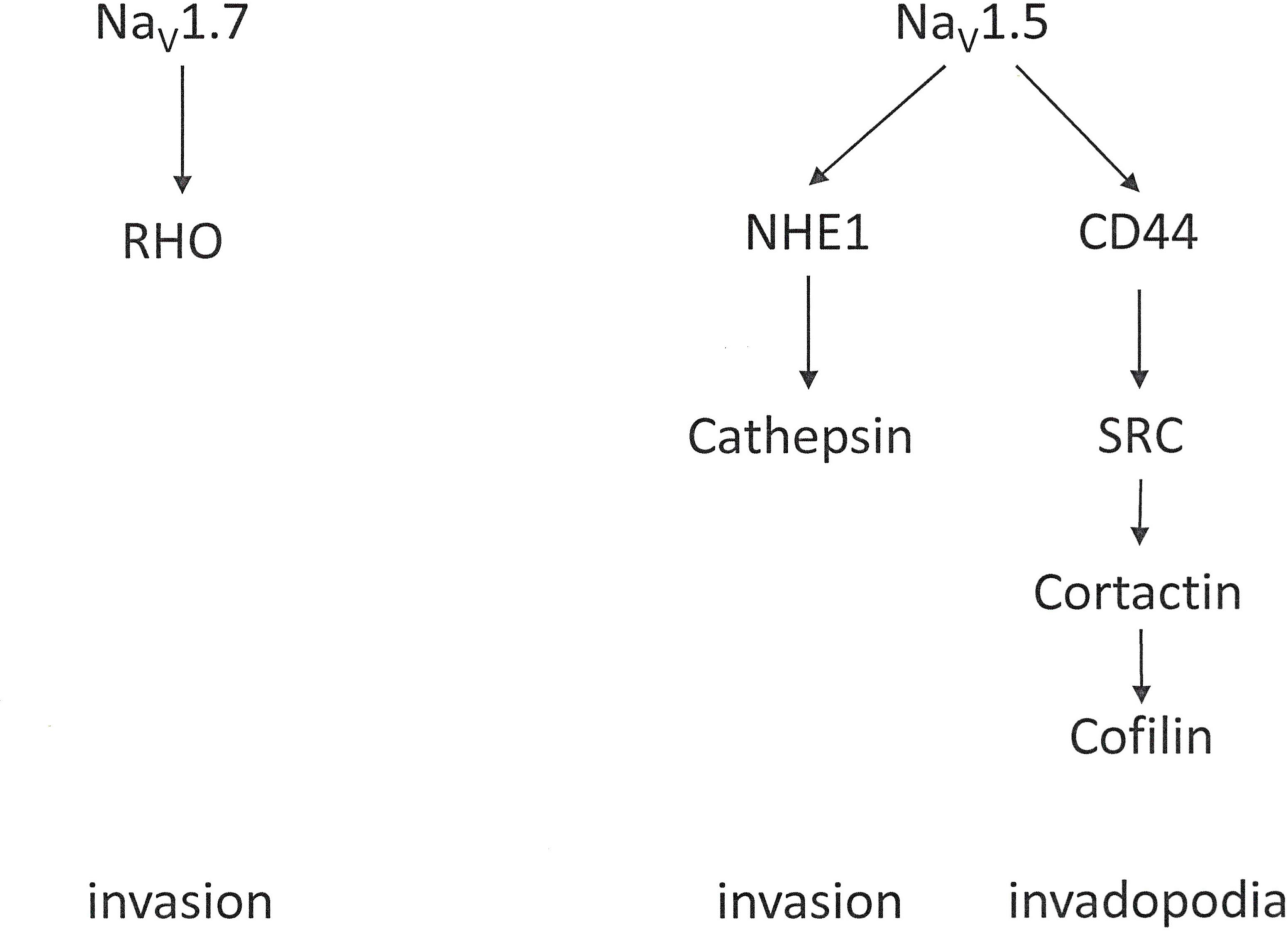
Voltage-gated sodium channels. Contributions by the voltage-gated sodium channels *Na_V_1.5* and *Na_V_1.7* to cell invasion.

Voltage-gated sodium channels contribute to the progression of various malignancies (118). They are widely expressed in invasive cancer cells with robust metastatic ability [4, 26] such as breast cancer (119–122), ovarian cancer (123, 124), prostate cancer  (125, 126), colon cancer (127) and non-small cell lung cancer (128). In this setting, *Na_V_1.5* and Na_V_1.7 are the most prevalent. *Na_V_1.5* is up-regulated in breast (129) and ovarian cancers, while *Na_V_1.7* is highly expressed in prostate (130), lung, and endometrial cancers. The functional expression of these channels contributes to cell invasion and tumor metastasis (118, 131, 132). In these cells, a sodium-current, imparted by the α subunits, up-regulates migration, invasion and metastasis (**Figure 7**). The β subunits, on the other hand, mediate cellular adhesion and process extension. The prevailing hypothesis suggests that voltage-gated Na^+^ channels are up-regulated in cancer, and their presence generally supports an invasive/metastatic phenotype.

Tumors often abnormally express voltage-gated Na^+^ channels as neonatal isoforms, which are not expressed, or only at a low level, in the corresponding healthy tissue. The extent of their abundance and their level of activity correlate with the aggressiveness of the disease and with the formation of metastases (133, 134). Expression of the *Na_V_1.5α* subunit indicates poor prognosis in breast cancers, suggesting that voltage-gated Na^+^ channels may be applied as prognostic markers for cancer progression (134). According to variant-specific antibody detection, the neonatal splice form of *Na_V_1.5* is specifically associated with breast cancer progression and enhanced metastatic potential (132). Furthermore, there is a strong correlation between neonatal *Na_V_1.5* expression and lymph node metastasis. Because up-regulation of neonatal *Na_V_1.5* occurs during metastatic progression in breast cancer, it could serve as a marker of the metastatic phenotype and also as a candidate therapeutic target (119).

In triple negative breast cancer, there is significant overlap in the expression of the β‐adrenergic receptor and voltage‐gated sodium channels. Short‐term (acute) administration of the β‐adrenergic receptor antagonist propranolol leads to a reduction in the peak current and a hastening of current inactivation. A protracted exposure to propranolol results in several changes in voltage-gated Na^+^ channel characteristics, comprising shifts in the current‐voltage relationship toward more negative potentials, steady‐state inactivation toward more negative potentials, and a slowing of the recovery from inactivation (135).

The Sodium-Hydrogen Exchanger 1 (*NHE1*) is ubiquitously expressed. It contributes to cell volume adjustments and intracellular pH homeostasis. In cancer, *NHE1* is up-regulated or over-expressed in several tumor types. There, it plays a profound role in progression and invasion by modulating the metabolic environment and cell intrusiveness. In breast cancer cells, movement independently of Actin polymerization and Integrin-dependent adhesion can arise through repetitive cycles of local *NHE1*-dependent swelling at the leading edge of the cell and shrinkage at the lagging end. This mechanism may underlie the rounded tumor cell migration that enables motility through a dense extracellular matrix without matrix degradation (136). The transporter *NHE1* is a downstream interacting partner to the α subunit of voltage-gated sodium channels. *NHE1*, activated by *VGSC*, can acidify the pericellular space, promoting extracellular proteolysis and invasiveness (137, 138). Subsequently, *SRC* kinase and invadopodia activity, which are promoted by activation of the Ca^2+^-dependent pathway also become involved in invasiveness (139).

Candidate Drug Treatments: With the contributions by sodium channels in migration, invasion and metastasis, there is potential for repurposing drugs with voltage-gated sodium channel blocking activities to achieve anti-invasion and anti-metastatic action (140). Sodium channel blockers are drugs, which impair the conduction of sodium ions through their cognate channels. These blockers are in clinical use for the treatment of cardiac arrhythmia (class I antiarrhythmic agents). Repurposing existing voltage-gated Na^+^ channel-blocking agents may provide a strategy to improve outcomes in metastatic disease (134). Pharmaceuticals that fulfill the required effects include lamotrigine, resveratrol, mexiletine, riluzole and the vasodilator flunarizine. Because these agents have shown anti-invasion and anti-metastatic activity, many of them have merit to be tested in clinical trials as adjunct treatments for solid tumors and as anti-metastatic agents (140).

Inhibiting voltage-gated sodium channels may decrease cancer spread and tumor progression, regardless of the channel subtypes or mechanisms of action, with tetrodotoxin (TTX) being the most extensively utilized ion channel blocker  (119, 137, 141). The number of lung metastases in a rodent model of prostate cancer is reduced by >40% after tetrodotoxin is given to suppress VGSC activity in primary tumors. This significantly increases the longevity of the animals, implying that VGSC activity supports prostate cancer metastasis *in vivo* (125).

*Nav1.5*-specific small interfering RNAs decrease the invasive capacity of colon cancer cells, suggesting that the *Nav1.5*-encoding gene SCN5A may regulate cell invasion-related signaling pathways (141). Neonatal and adult splice variants of *Na_V_1.5* are expressed by metastatic colon and breast cancer cells. The inhibition of voltage-activated sodium channels on circulating metastatic cancer cells may constitute an efficacious approach for perioperative patients. In metastatic colon cancer cells, the local anesthetic drug ropivacaine blocks the activity of the *NaV1.5* channel and the invasion  (127).

The selective inhibitor of the persistent cardiac Na^+^ current, ranolazine, is in clinical use for treating chronic stable angina. It also reduces the sodium-dependent calcium overload that occurs in cardiomyocytes during ischemia. Ranolazine suppresses the invasiveness of breast and prostate cancer cells (142). β‐adrenergic receptor and voltage-gated Na^+^ channel are functionally coupled in some breast cancer cells. The β blocker propranolol significantly decreases total neonatal *Na_V_1.5* protein expression, which is the most abundantly expressed VGSC subtype present in these cells. Propranolol also has direct blocking activity on the voltage-gated Na^+^ channel. In breast cancer cells, propranolol, ranolazine and their combination inhibit motility and invasion. Yet, there is no synergy in the pharmacological combination of these agents. On the cellular level, the anti-metastatic effects of ranolazine and propranolol are not additive (135).

In prostate cancer, the synthetic sodium channel blockers S0154 and S0161 show anti-cancer and anti-metastatic effects. Both compounds increase the intracellular level of sodium. They significantly inhibit the protein expression of two α subunits in voltage-gated Na^+^ channels (*Na_V_1.6* and *Na_V_1.7*) and cause G_2_/M phase cell cycle arrest, while having no or minor effects on apoptosis. Low concentrations of S0154 and S0161 decrease the glucose uptake by prostate cancer cells. These agents also inhibit the proliferation of prostate cancer cells and suppress their invasion. It is implied that S0154 and S0161 may have anti-cancer and anti-metastasis effects against prostate cancer cells, which substantiates their further development as prospective therapeutic compounds (118).

Prostate cancer cells over-express voltage-gated sodium channels, and their metastatic behaviors are associated with these channels. Naringenin counteracts prostate cancer metastasis by blocking VGSCs. While high concentrations of the drug inhibit cell proliferation, low concentrations decrease the movement of prostate cancer cells. Naringenin has blocking activity (directly or indirectly) on voltage-gated sodium channels that are encoded by the *SCN9A* gene. It also displays inhibitory effects on cell motility by lowering *SCN9A* gene expression on the mRNA level (143).

Eicosapentaenoic acid inhibits voltage-gated sodium channels and suppresses invasiveness in prostate cancer cells. Exposure of prostate cancer cells to eicosapentaenoic acid produces a fast and concentration-dependent reduction of inward Na^+^ currents. After extended exposure, the eicosapentaenoic acid content of cell lipids increases time-dependently, while the arachidonic acid content decreases (126).

Anti-epileptic drugs, such as phenytoin, carbamazepine and valproate, have emerged as prospectively beneficial for anti-metastatic purposes (140). Phenytoin has been reported to suppress both transient and sustained Na^+^ flow in breast cancer cells. Within therapeutic concentrations, it may prevent the migration and invasion of these cells. However, it has no effect on non-invasive breast tumor cells that do not produce Na^+^ currents (144). Phenytoin suppresses breast tumor development and metastasis *in vivo* and reduces metastatic breast cancer cell motility and invasion *in vitro* (144, 145). Therefore, phenytoin could be a candidate treatment option for metastatic breast cancer.

## Chloride

General: Chloride channels comprise voltage-gated and ligand-gated channels. Based on sequence homology, these classes of channels fall into the groups of the CLC family, the epithelial chloride channel (E-CIC) family, the chloride intracellular ion channel (CLIC) family, and the cystic fibrosis transmembrane conductance regulator (*CFTR*). The functions of chloride channels cover a scope from ion homeostasis to cell volume maintenance and regulation of excitable cells (146).

Functions in metastasis: The chloride channels that mediate voltage-activated currents and contribute to glioma cell migration may be the same channels that effectuate volume-sensitive chloride currents. *ClC-3* is the major volume- and voltage-activated chloride channel in malignant cells, which contributes to volume regulation and migration. Physiologically, *CIC-3* is expressed abundantly in neurons.

Candidate Drug Treatments: Cl^-^ currents are sensitive to inhibition by the 36-amino acid peptide chlorotoxin, a pharmacologic agent that is purified from the venom of the deathstalker scorpion. Other channel inhibitors include 4,4’-diisothiocyano-2,2’-stilbenedisulfonic acid (DIDS), tamoxifen, Zn^2+^, and Natriuretic Peptide B (NPPB). At rest, glioma cells have a basal chloride current. Blockers of volume-activated chloride currents may boost their membrane resistance (147). In clinical trials, inhibition of chloride channels has displayed potential to serve as therapeutic approach for the treatment of gliomas (69).

## NKCC

General: Ubiquitously expressed, voltage-activated ion channels contribute to facilitating cell migration and invasion. The concerted release of osmotically active ions supports volume changes, mostly involving K^+^ and Cl^−^. These ions adjust a cell’s shape and size by crossing the plasma membrane in conjunction with obligated water. Speedy cell shrinkage is effectuated by their efflux through ion channels along with the obligated water. Calcium-activated potassium channels and voltage-gated chloride channels are to a large extent accountable for effluxing KCl and fostering hydrodynamic volume changes. It is essential to establish and maintain suitable ionic gradients through the activity of ion transport systems (148). The sodium-potassium-chloride cotransporters (NKCC) aid in the secondary active transport of these ions. *NKCC1* (Sodium-Potassium-Chloride Cotransporter Isoform-1) and *NKCC2* are encoded by the genes *SLC12A2* and *SLC12A1* respectively.

Functions in Metastasis: In diverse cancers, various potassium or sodium channels may function in concert with diverse chloride channels, in result to support changes in cell volume (69). Malignant gliomas spread metastases into peritumoral areas of the brain by infiltrative cell migration. These transformed cells encounter restricted spaces and may be able to adjust their shape for accommodating to narrow extracellular spaces. Through their Na^+^-K^+^-2Cl^-^ cotransporters, they actively accumulate K^+^ and Cl^-^ (*NKCC1* enables the major pathway for chloride accumulation in glioma cells), thus establishing a gradient for KCl efflux. The channels localize to the leading edge of invading processes, and their inhibition partially inhibits migration. A loss of *NKCC1* function manifests, when cells have to undergo volume changes during migration (69, 148). *NKCC1* is present in meningiomas of all grades. The invasion of meningioma cells into the dura may contribute to the high rates of recurrence. Implicated in the invasion are ion channels that affect cell shape and movement.

**Figure 8:**
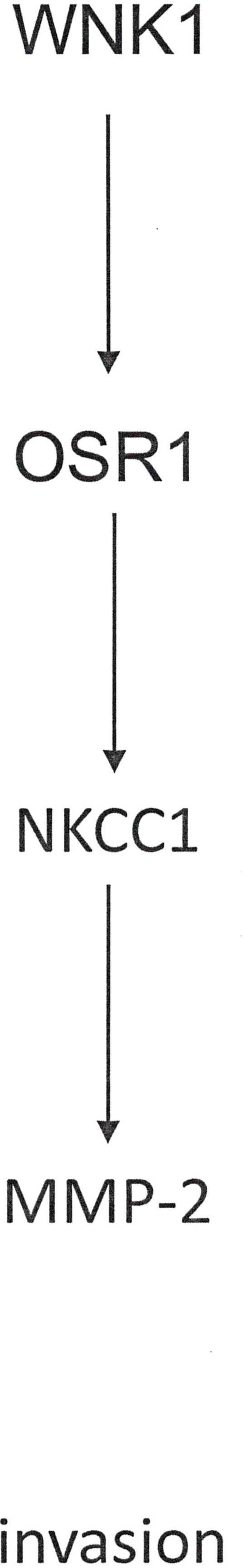
Support of cancer invasion by NKCC channels. The pathway displayed is one mechanism to induce Matrix Metalloproteinases and facilitate invasion.

The primary culprit for the high mortality of hepatocellular carcinoma is metastasis. By immunohistochemistry, *NKCC1* expression is related to poor differentiation and microvascular invasion. In hepatocellular carcinoma cell lines, *NKCC1* is up-regulated in the highly invasive cells. The channel is necessary and sufficient for proliferation and invasion abilities by this cancer. *NKCC1* promotes invasiveness *via* MMP-2 activity, and the *WNK1*/*OSR1*/*NKCC1* signal pathway (**Figure 8**) might play roles in hepatocellular carcinoma metastasis (149).

Candidate Drug Treatments: The pharmacologic suppression of *NKCC1* with the loop diuretic bumetanide may decrease neoplastic migration (69). Because *NKCC1* plays a role in hepatocellular carcinoma metastasis, it can serve as a candidate drug target to inhibit dissemination. Consistently, using bumetanide to block *NKCC1* activity attenuates the proliferation and invasion propensities of hepatocellular carcinoma cells and limits tumor growth (149). Malignant gliomas metastasize throughout the brain with *NKCC1* activating the major pathway for Cl^−^ accumulation in these cells. Pharmacologic inhibition using bumetanide partially inhibits migration (148). In these glioma cells, suitable ionic gradients need to be established through the activity of ion transport systems, such as *NKCC1*. While a loss of *NKCC1* function does not compromise cell motility in two-dimensional assays that lack spatial constraints, it manifests when cells have to undergo volume changes during locomotion. Collectively implied is the consideration of bumetanide as an adjuvant therapy for patients with gliomas of high-grade (148).

The cotransporter *NKCC1* serves to accumulate intracellular chloride to high concentrations. Thus, it establishes an outward directed Cl^-^ gradient, which enables invading glioma cells to utilize chloride as an osmotically active anion. The blocking of Cl^-^ channels retards changes in cell volume and compromises cancer cell invasion. These mechanisms have prompted the clinical evaluation of the chloride channel blocking peptide chlorotoxin for the treatment of malignant glioma. The agent displays strong tumor selectivity, it is well tolerated and has moved into phase II clinical trials (150).

## Water

General: Ion channels may facilitate locomotion through modulations of the cell volume, which is particularly necessary when cells move through narrow spaces. At the lamellipodium, Aquaporins mediate water influx. In that compartment, Aquaporin activity increases, when an accumulation of depolymerized Actin in conjunction with osmotically active ions induce focal intracellular hypertonicity. The resulting osmotic water influx causes swelling. The upsurge in cytoplasmic water content can consecutively trigger greater elevated hydrostatic pressure, thus inducing membrane protrusion at the leading edge (151). Through these mechanisms, Aquaporins enable plasticity at the leading edge and move the cell forward *via* elevations in intracellular hydrostatic pressure. Ion channels and Aquaporins operate in coordination to modify cellular volume, propel the leading edge forward, and augment dynamic shape changes.

## Various Synergies Among Ion-Regulating Mechanisms

Ion channels and transporters in cooperation with water-permeable Aquaporins contribute to cell motility by regulating the local Ca^2+^, pH and volume homeostasis. Aquaporins push the outgrowth of lamellipodia or invadopodia. They also may control the number of Integrin β_1_ receptors in the plasma membrane. The multiplicity of interacting ion channels and transporters, in conjunction with the associated signaling events, holds potential as targets for anti-cancer agents that are directed at preventing metastasis (152).

Cell migration, an important element of the metastatic cascade, requires a coordinated action of ion transporters and channels with cytoskeletal components. Ion transport proteins are often over-expressed or activated in cancer. In conjunction with Aquaporins, they contribute to cancer cell migration and invasion, including through the induction of local volume changes or through the modulation of Ca^2+^ signaling (21). Channels involved in cancer cell motility are commonly regulated by calcium signaling. The most likely basis for this is the coupling of extracellular stimuli to cell migration. Voltage-gated chloride channels and calcium-activated potassium channels are responsible to a large extent for effluxing potassium chloride and supporting hydrodynamic volume changes (69).

Calcium is a major mediator of migratory activity. The increased expression of K^+^ channels on transformed cells can lead to a more negative membrane potential, which constitutes a substantial driving force for calcium entry through *ORAI1*. *K_V_10.1* and *ORAI1* are both expressed in lymph node metastases from breast cancer. While the chloride concentration gradient is the primary driver in controlling the osmotic pressure, a potassium flow is also required to neutralize any arising charge imbalance. Chloride channel inhibitors are in clinical trials for patients with glioma (150).

Potassium channel function can interact with other ions. Where a substantive contribution to volume regulation and migration is made by chloride channels, there must concomitantly be the permeation of a cation to maintain electroneutrality. Potassium channels support the locomotion of malignant cells by allowing potassium, which is accumulated inside the cells through the Na^+^-K^+^-ATPase, to exit these cells and support volume regulation. Hence, two kinds of channels, those for K^+^ and those for Cl^−^, may work in concert. They enable the efflux of potassium and chloride ions, consecutively prompting osmotic water release and cytoplasmic condensation in migrating cancer cells. In glioma cells, lipid rafts contain Cl^−^ channels, and they colocalize with BK channels on the invadapodia. Migrating glioma cells encounter tight spaces and may be able to adjust their shape for accommodating to narrow extracellular environments. Calcium-activated potassium channels and voltage-gated chloride channels are the main mediators for the efflux of KCl. Thus, they promote hydrodynamic volume changes. Some of these channels are active in cancer-type specific manners. Across diverse types of cancers, various potassium or even sodium channels may function in coordination with diverse chloride channels to support comparable volume changes (69).

K^+^ channels may contribute to aberrant tumor growth. Their exposure to channel inhibitors often results in growth arrest. There is a role for potassium and chloride channels in primary brain tumors, specifically gliomas, where the coordinated activity of these channels supports cell invasion, resulting in the formation of brain metastasis. At the invading processes of tumor cells, calcium-activated potassium channels colocalize with *ClC-3* chloride channels. An expeditious shrinkage of the leading edge is effectuated by a rise in intracellular calcium, which causes these channels to activate and release potassium and chloride ions together with obligated water. This process enables the invasion of the tumor cell into the narrow brain spaces. The cotransporter *NKCC1* accrues intracellular chloride to elevated levels. This establishes an outward directed gradient for chloride ions and enables glioma cells to utilize these charged particles as osmotically active anions during invasion. Blocking of chloride channels, importantly, slows down cell volume changes, and limits tumor cell invasion. These mechanisms have prompted the clinical evaluation of the chloride channel blocker chlorotoxin as a treatment for malignant glioma. The results indicate unexpected tumor selectivity for this agent. The experimental therapeutic is well tolerated and has entered evaluation advanced clinical trials. It is likely that other metastatic cancers engage Cl^-^ and K^+^ channels in similar fashions, and lessons learned from the glioma studies may pave the way towards the development of therapeutics that block ion channels (150).

The high rate of recurrence by meningiomas may be caused, in part, by invasion of the dura. Ion channels that affect motion and shape of the cells contribute to cancer invasion. The combined sodium-potassium-chloride cotransporter 1 (*NKCC1*) is present in essentially all World Health Organization grade I, and most grade II and grade Ill meningiomas. There is extensive *NKCC1* but no Aquaporin 1 (*AQP1*) presence in the arachnoid granulation cells, and widespread immunohistochemical staining of *NKCC1* in meningioma cells and in capillaries. In tumors with invasion of the dura or bone/soft tissue, immunoreactivity for *NKCC1* is present in all cases of invading cells and in the majority of their capillaries. *AQP1* is also present in most meningioma cells and in all capillaries. The marker is positive in cells and capillaries, which invade the dura or the bone. In all subtypes of meningiomas, this phenomenon is extensive. It is implied that *AQP1* and *NKCC1* contribute to meningioma biology and invasion (153).

Ion transporters and channels play roles in cell motility. They do so by passing or transporting ions that are essential for local Ca^2+^, pH and – in synergy with Aquaporin-mediated water movement – volume homeostasis. They, or their auxiliary subunits, furthermore exhibit non-conducting activities. They may exert kinase activity, which phosphorylates cytoskeletal components or their associates. They may also engage intracellular signaling processes *via* generation of local pH-nanodomains, *via* calcium permeation, or *via* acting as final downstream effectors. A substantial number of transporters and channels locate to focal adhesions, where they interact directly or indirectly with Integrins, with elements of the cytoskeleton, or with proteins of the extracellular matrix. Aquaporins drive the outgrowth of lamellipodia or invadopodia. They can control the number of Integrins β_1_ in the plasma membrane (152).

## Discussion and Conclusions

Metastases are characterized by a core program of gene expression that distinguishes them from their sites of origin as well as from their target sites. In addition to increased oxidative metabolism, reduced extracellular matrix interactions and vascularization/tissue remodeling, the ion homeostasis is extensively skewed. In principle, this alteration has potential for therapeutic targeting. Yet, three factors have hampered the pursuit of this goal.

- The ionic changes have been difficult to characterize. They may entail multiple adaptations, because an imbalance in one charged particle can secondarily impact others. A level of charge balance needs to be maintained.
- While the phenomenon of ionic skewing is common to all metastases, their exact modalities may differ among diverse malignancies. This is the case for zinc, up-regulation of which arises in breast and ovarian cancers. By contrast, the healthy prostate contains high levels of zinc. Its transformation and progression are characterized by lowered zinc levels. Similarly, voltage-gated potassium channels are elevated in some cancers and reduced in others.
- The maintenance of non-equilibrium conditions with transmembrane gradients of ions is common to all cells, so that any drug-targeting of the ionic balance in cancer cells will lack selectivity and entail risks for adverse effects, caused by the disturbance of healthy cells. How much of a problem this is remains to be investigated. Many modulators of ionic homeostasis are in clinical use for other diseases, where they have proven to be safe and efficacious.

The growing literature on ionic imbalance in cancer progression has revealed some changes as being common to multiple malignancies. Their identification as promising drug targets has led to early-stage clinical trials for several chelators or ion channel blockers (the supplementation of down-regulated ions has found less interest). Thus far, the levels of success for these agents have been modest, possibly because monotherapy rarely achieves efficacy in cancer. If partially successful in trials, these drugs will need to be incorporated into combination treatments.

## Author Contributions

GW wrote the review. GF did the referencing and substantive editing. Both authors have searched the literature. All authors contributed to the article and approved the submitted version.

## Funding

This research was supported by NIH grant CA224104 and a Steven Goldman Memorial Grant to GW.

## Conflict of Interest

The authors declare that the research was conducted in the absence of any commercial or financial relationships that could be construed as a potential conflict of interest.

## Publisher’s Note

All claims expressed in this article are solely those of the authors and do not necessarily represent those of their affiliated organizations, or those of the publisher, the editors and the reviewers. Any product that may be evaluated in this article, or claim that may be made by its manufacturer, is not guaranteed or endorsed by the publisher.

## References

[1] Loeb LA. A Mutator Phenotype in Cancer. Cancer Res (2001) 61(8):3230–9. 11309271

[2] Sun R Hu Z Curtis C. Big Bang Tumor Growth and Clonal Evolution. Cold Spring Harbor Perspect Med (2018) 8(5):a028381. doi: 10.1101/cshperspect.a028381 PMC5932575 28710260

[3] Vendramin R Litchfield K Swanton C. Cancer Evolution: Darwin and Beyond. EMBO J (2021) 40(18):e108389. doi: 10.15252/embj.2021108389 34459009 PMC8441388

[4] Weber GF Ashkar S. Stress Response Genes: The Genes That Make Cancer Metastasize. J Mol Med (Berl) (2000) 78(8):404–8. doi: 10.1007/s001090000138 11097109

[5] Hartung F Wang Y Aronow B Weber GF. A Core Program of Gene Expression Characterizes Cancer Metastases. Oncotarget (2017) 8(60):102161–75. doi: 10.18632/oncotarget.22240 PMC5731943 29254233

[6] Hartung F Patil A Meshram RJ Weber GF. Gene Expression Signatures of Site-Specificity in Cancer Metastases. Clin Exp Metastasis (2020) 37(1):159–71. doi: 10.1007/s10585-019-09995-w 31555944

[7] He B Mirza M Weber GF. An Osteopontin Splice Variant Induces Anchorage Independence in Human Breast Cancer Cells. Oncogene (2006) 25(15):2192–202. doi: 10.1038/sj.onc.1209248 16288209

[8] Weber GF. Molecular Analysis of a Recurrent Sarcoma Identifies a Mutation in FAF1. Sarcoma (2015) 2015:839182. doi: 10.1155/2015/839182 25861239 PMC4377510

[9] Pardo LA Stuhmer W. The Roles of K(+) Channels in Cancer. Nat Rev Cancer (2014) 14(1):39–48. doi: 10.1038/nrc3635 24336491

[10] Kaplan RN Riba RD Zacharoulis S Bramley AH Vincent L Costa C. VEGFR1-Positive Haematopoietic Bone Marrow Progenitors Initiate the Pre-Metastatic Niche. Nature (2005) 438(7069):820–7. doi: 10.1038/nature04186 PMC2945882 16341007

[11] Cotran RS Kumar V Collins T. Robbins Pathologic Basis of Disease. Philadelphia, PA W.B. Saunders Company (1999).

[12] Zhang G He B Weber GF. Growth Factor Signaling Induces Metastasis Genes in Transformed Cells: Molecular Connection Between Akt Kinase and Osteopontin in Breast Cancer. Mol Cell Biol (2003) 23(18):6507–19. doi: 10.1128/mcb.23.18.6507-6519.2003 PMC193717 12944477

[13] Weber GF. Molecular Mechanisms of Metastasis. Cancer Lett (2008) 270(2):181–90. doi: 10.1016/j.canlet.2008.04.030 18522865

[14] Azimi I Monteith GR. Plasma Membrane Ion Channels and Epithelial to Mesenchymal Transition in Cancer Cells. Endocr-Related Cancer (2016) 23(11):R517–25. doi: 10.1530/erc-16-0334 27619258

[15] Liu L Wu N Wang Y Zhang X Xia B Tang J. TRPM7 Promotes the Epithelial-Mesenchymal Transition in Ovarian Cancer Through the Calcium-Related PI3K / AKT Oncogenic Signaling. J Exp Clin Cancer Res CR (2019) 38(1):106. doi: 10.1186/s13046-019-1061-y 30819230 PMC6396458

[16] Azimi I Robitaille M Armitage K So CL Milevskiy MJG Northwood K. Activation of the Ion Channel TRPV4 Induces Epithelial to Mesenchymal Transition in Breast Cancer Cells. Int J Mol Sci (2020) 21(24):9417. doi: 10.3390/ijms21249417 PMC7764818 33322037

[17] Kärki T Rajakylä EK Acheva A Tojkander S. TRPV6 Calcium Channel Directs Homeostasis of the Mammary Epithelial Sheets and Controls Epithelial Mesenchymal Transition. Sci Rep (2020) 10(1):14683. doi: 10.1038/s41598-020-71645-z 32895467 PMC7477193

[18] Paoli P Giannoni E Chiarugi P. Anoikis Molecular Pathways and Its Role in Cancer Progression. Biochim Biophys Acta (2013) 1833(12):3481–98. doi: 10.1016/j.bbamcr.2013.06.026 23830918

[19] Yu SP Canzoniero LM Choi DW. Ion Homeostasis and Apoptosis. Curr Opin Cell Biol (2001) 13(4):405–11. doi: 10.1016/s0955-0674(00)00228-3 11454444

[20] Yang M Brackenbury WJ. Membrane Potential and Cancer Progression. Front Physiol (2013) 4:185. doi: 10.3389/fphys.2013.00185 23882223 PMC3713347

[21] Schwab A Stock C. Ion Channels and Transporters in Tumour Cell Migration and Invasion. Philos Trans R Soc London Ser B Biol Sci (2014) 369(1638):20130102. doi: 10.1098/rstb.2013.0102 24493750 PMC3917356

[22] Prevarskaya N Skryma R Shuba Y. Calcium in Tumour Metastasis: New Roles for Known Actors. Nat Rev Cancer (2011) 11(8):609–18. doi: 10.1038/nrc3105 21779011

[23] Jacquemet G Baghirov H Georgiadou M Sihto H Peuhu E Cettour-Janet P. L-Type Calcium Channels Regulate Filopodia Stability and Cancer Cell Invasion Downstream of Integrin Signalling. Nat Commun (2016) 7:13297. doi: 10.1038/ncomms13297 27910855 PMC5146291

[24] Yang S Zhang JJ Huang XY. Orai1 and STIM1 are Critical for Breast Tumor Cell Migration and Metastasis. Cancer Cell (2009) 15(2):124–34. doi: 10.1016/j.ccr.2008.12.019 19185847

[25] Kang Q Peng X Li X Hu D Wen G Wei Z. Calcium Channel Protein ORAI1 Mediates TGF-β Induced Epithelial-to-Mesenchymal Transition in Colorectal Cancer Cells. Front Oncol (2021) 11:649476. doi: 10.3389/fonc.2021.649476 34055617 PMC8149897

[26] Guo Y Zhu J Wang X Li R Jiang K Chen S. Orai1 Promotes Osteosarcoma Metastasis by Activating the Ras-Rac1-WAVE2 Signaling Pathway. Med Sci monitor Int Med J Exp Clin Res (2019) 25:9227–36. doi: 10.12659/msm.919594 PMC6909920 31796725

[27] Davis FM Azimi I Faville RA Peters AA Jalink K Putney JW Jr. Induction of Epithelial-Mesenchymal Transition (EMT) in Breast Cancer Cells Is Calcium Signal Dependent. Oncogene (2014) 33(18):2307–16. doi: 10.1038/onc.2013.187 PMC3917976 23686305

[28] Stewart TA Azimi I Thompson EW Roberts-Thomson SJ Monteith GR. A Role for Calcium in the Regulation of ATP-Binding Cassette, Sub-Family C, Member 3 (ABCC3) Gene Expression in a Model of Epidermal Growth Factor-Mediated Breast Cancer Epithelial-Mesenchymal Transition. Biochem Biophys Res Commun (2015) 458(3):509–14. doi: 10.1016/j.bbrc.2015.01.141 25666946

[29] Pratt SJP Hernández-Ochoa EO Lee RM Ory EC Lyons JS Joca HC. Real-Time Scratch Assay Reveals Mechanisms of Early Calcium Signaling in Breast Cancer Cells in Response to Wounding. Oncotarget (2018) 9(38):25008–24. doi: 10.18632/oncotarget.25186 PMC5982755 29861849

[30] Comes N Serrano-Albarrás A Capera J Serrano-Novillo C Condom E Ramón YCS. Involvement of Potassium Channels in the Progression of Cancer to a More Malignant Phenotype. Biochim Biophys Acta (2015) 1848(10 Pt B):2477–92. doi: 10.1016/j.bbamem.2014.12.008 25517985

[31] Becchetti A Arcangeli A. Integrins and Ion Channels in Cell Migration: Implications for Neuronal Development, Wound Healing and Metastatic Spread. Adv Exp Med Biol (2010) 674:107–23. doi: 10.1007/978-1-4419-6066-5_10 20549944

[32] Schwab A Fabian A Hanley PJ Stock C. Role of Ion Channels and Transporters in Cell Migration. Physiol Rev (2012) 92(4):1865–913. doi: 10.1152/physrev.00018.2011 23073633

[33] Wei JF Wei L Zhou X Lu ZY Francis K Hu XY. Formation of Kv2.1-FAK Complex as a Mechanism of FAK Activation, Cell Polarization and Enhanced Motility. J Cell Physiol (2008) 217(2):544–57. doi: 10.1002/jcp.21530 PMC2562431 18615577

[34] Cheng L Yung A Covarrubias M Radice GL. Cortactin is Required for N-Cadherin Regulation of Kv1.5 Channel Function. J Biol Chem (2011) 286(23):20478–89. doi: 10.1074/jbc.M111.218560 PMC3121477 21507952

[35] Hattan D Nesti E Cachero TG Morielli AD. Tyrosine Phosphorylation of Kv1.2 Modulates Its Interaction With the Actin-Binding Protein Cortactin. J Biol Chem (2002) 277(41):38596–606. doi: 10.1074/jbc.M205005200 12151401

[36] Williams MR Markey JC Doczi MA Morielli AD. An Essential Role for Cortactin in the Modulation of the Potassium Channel Kv1.2. Proc Natl Acad Sci USA (2007) 104(44):17412–7. doi: 10.1073/pnas.0703865104 PMC2077270 17959782

[37] Breuer EK Fukushiro-Lopes D Dalheim A Burnette M Zartman J Kaja S. Potassium Channel Activity Controls Breast Cancer Metastasis by Affecting β-Catenin Signaling. Cell Death Dis (2019) 10(3):180. doi: 10.1038/s41419-019-1429-0 30792401 PMC6385342

[38] Bielanska J Hernández-Losa J Moline T Somoza R Ramón y Cajal S Condom E. Differential Expression of Kv1.3 and Kv1.5 Voltage-Dependent K+ Channels in Human Skeletal Muscle Sarcomas. Cancer Invest (2012) 30(3):203–8. doi: 10.3109/07357907.2012.654872 22360360

[39] Laniado ME Fraser SP Djamgoz MB. Voltage-Gated K(+) Channel Activity in Human Prostate Cancer Cell Lines of Markedly Different Metastatic Potential: Distinguishing Characteristics of PC-3 and LNCaP Cells. Prostate (2001) 46(4):262–74. doi: 10.1002/1097-0045(20010301)46:4<262::aid-pros1032>3.0.co;2-f 11241548

[40] Abdul M Santo A Hoosein N. Activity of Potassium Channel-Blockers in Breast Cancer. Anticancer Res (2003) 23(4):3347–51. 12926074

[41] Bielanska J Hernández-Losa J Pérez-Verdaguer M Moline T Somoza R Ramón YCS. Voltage-Dependent Potassium Channels Kv1.3 and Kv1.5 in Human Cancer. Curr Cancer Drug Targets (2009) 9(8):904–14. doi: 10.2174/156800909790192400 20025600

[42] Jang SH Kang KS Ryu PD Lee SY. Kv1.3 Voltage-Gated K(+) Channel Subunit as a Potential Diagnostic Marker and Therapeutic Target for Breast Cancer. BMB Rep (2009) 42(8):535–9. doi: 10.5483/bmbrep.2009.42.8.535 19712592

[43] Brevet M Haren N Sevestre H Merviel P Ouadid-Ahidouch H. DNA Methylation of K(v)1.3 Potassium Channel Gene Promoter Is Associated With Poorly Differentiated Breast Adenocarcinoma. Cell Physiol Biochem Int J Exp Cell Physiol Biochem Pharmacol (2009) 24(1-2):25–32. doi: 10.1159/000227810 19590190

[44] Bielanska J Hernández-Losa J Moline T Somoza R Ramón YCS Condom E. Increased Voltage-Dependent K(+) Channel Kv1.3 and Kv1.5 Expression Correlates With Leiomyosarcoma Aggressiveness. Oncol Lett (2012) 4(2):227–30. doi: 10.3892/ol.2012.718 PMC3402732 22844358

[45] Preussat K Beetz C Schrey M Kraft R Wölfl S Kalff R. Expression of Voltage-Gated Potassium Channels Kv1.3 and Kv1.5 in Human Gliomas. Neurosci Lett (2003) 346(1-2):33–6. doi: 10.1016/s0304-3940(03)00562-7 12850541

[46] Downie BR Sánchez A Knötgen H Contreras-Jurado C Gymnopoulos M Weber C. Eag1 Expression Interferes With Hypoxia Homeostasis and Induces Angiogenesis in Tumors. J Biol Chem (2008) 283(52):36234–40. doi: 10.1074/jbc.M801830200 PMC2606018 18927085

[47] Agarwal JR Griesinger F Stühmer W Pardo LA. The Potassium Channel Ether À Go-Go Is a Novel Prognostic Factor With Functional Relevance in Acute Myeloid Leukemia. Mol Cancer (2010) 9:18. doi: 10.1186/1476-4598-9-18 20105281 PMC2835655

[48] Hammadi M Chopin V Matifat F Dhennin-Duthille I Chasseraud M Sevestre H. Human Ether À-Gogo K(+) Channel 1 (Heag1) Regulates MDA-MB-231 Breast Cancer Cell Migration Through Orai1-Dependent Calcium Entry. J Cell Physiol (2012) 227(12):3837–46. doi: 10.1002/jcp.24095 22495877

[49] Becchetti A Crescioli S Zanieri F Petroni G Mercatelli R Coppola S. The Conformational State of Herg1 Channels Determines Integrin Association, Downstream Signaling, and Cancer Progression. Sci Signaling (2017) 10(473):eaaf3236. doi: 10.1126/scisignal.aaf3236 28377405

[50] Neylon CB Avdonin PV Larsen MA Bobik A. Rat Aortic Smooth Muscle Cells Expressing Charybdotoxin-Sensitive Potassium Channels Exhibit Enhanced Proliferative Responses. Clin Exp Pharmacol Physiol (1994) 21(2):117–20. doi: 10.1111/j.1440-1681.1994.tb02477.x 7518756

[51] Wiecha J Münz B Wu Y Noll T Tillmanns H Waldecker B. Blockade of Ca2+-Activated K+ Channels Inhibits Proliferation of Human Endothelial Cells Induced by Basic Fibroblast Growth Factor. J Vasc Res (1998) 35(5):363–71. doi: 10.1159/000025606 9789117

[52] Faehling M Koch ED Raithel J Trischler G Waltenberger J. Vascular Endothelial Growth Factor-A Activates Ca2+ -Activated K+ Channels in Human Endothelial Cells in Culture. Int J Biochem Cell Biol (2001) 33(4):337–46. doi: 10.1016/s1357-2725(01)00021-8 11312104

[53] Pillozzi S Brizzi MF Bernabei PA Bartolozzi B Caporale R Basile V. VEGFR-1 (FLT-1), Beta1 Integrin, and hERG K+ Channel for a Macromolecular Signaling Complex in Acute Myeloid Leukemia: Role in Cell Migration and Clinical Outcome. Blood (2007) 110(4):1238–50. doi: 10.1182/blood-2006-02-003772 17420287

[54] Masi A Becchetti A Restano-Cassulini R Polvani S Hofmann G Buccoliero AM. Herg1 Channels are Overexpressed in Glioblastoma Multiforme and Modulate VEGF Secretion in Glioblastoma Cell Lines. Br J Cancer (2005) 93(7):781–92. doi: 10.1038/sj.bjc.6602775 PMC2361632 16175187

[55] Menéndez ST Villaronga MA Rodrigo JP Alvarez-Teijeiro S García-Carracedo D Urdinguio RG. Frequent Aberrant Expression of the Human Ether À Go-Go (Heag1) Potassium Channel in Head and Neck Cancer: Pathobiological Mechanisms and Clinical Implications. J Mol Med (Berlin Germany) (2012) 90(10):1173–84. doi: 10.1007/s00109-012-0893-0 22466864

[56] Ding XW Luo HS Jin X Yan JJ Ai YW. Aberrant Expression of Eag1 Potassium Channels in Gastric Cancer Patients and Cell Lines. Med Oncol (Northwood London England) (2007) 24(3):345–50. doi: 10.1007/s12032-007-0015-y 17873312

[57] Ding XW Yan JJ An P Lü P Luo HS. Aberrant Expression of Ether À Go-Go Potassium Channel in Colorectal Cancer Patients and Cell Lines. World J Gastroenterol (2007) 13(8):1257–61. doi: 10.3748/wjg.v13.i8.1257 PMC4147004 17451210

[58] Lastraioli E Guasti L Crociani O Polvani S Hofmann G Witchel H. Herg1 Gene and HERG1 Protein Are Overexpressed in Colorectal Cancers and Regulate Cell Invasion of Tumor Cells. Cancer Res (2004) 64(2):606–11. doi: 10.1158/0008-5472.can-03-2360 14744775

[59] Crociani O Zanieri F Pillozzi S Lastraioli E Stefanini M Fiore A. Herg1 Channels Modulate Integrin Signaling to Trigger Angiogenesis and Tumor Progression in Colorectal Cancer. Sci Rep (2013) 3:3308. doi: 10.1038/srep03308 24270902 PMC3839040

[60] Khaitan D Sankpal UT Weksler B Meister EA Romero IA Couraud PO. Role of KCNMA1 Gene in Breast Cancer Invasion and Metastasis to Brain. BMC Cancer (2009) 9:258. doi: 10.1186/1471-2407-9-258 19640305 PMC2727533

[61] Ransom CB Liu X Sontheimer H. BK Channels in Human Glioma Cells Have Enhanced Calcium Sensitivity. Glia (2002) 38(4):281–91. doi: 10.1002/glia.10064 12007141

[62] Sciaccaluga M Fioretti B Catacuzzeno L Pagani F Bertollini C Rosito M. CXCL12-Induced Glioblastoma Cell Migration Requires Intermediate Conductance Ca2+-Activated K+ Channel Activity. Am J Physiol Cell Physiol (2010) 299(1):C175–84. doi: 10.1152/ajpcell.00344.2009 20392929

[63] Catacuzzeno L Fioretti B Franciolini F. Expression and Role of the Intermediate-Conductance Calcium-Activated Potassium Channel KCa3.1 in Glioblastoma. J Signal Transduction (2012) 2012:421564. doi: 10.1155/2012/421564 PMC3362965 22675627

[64] Gómez-Varela D Zwick-Wallasch E Knötgen H Sánchez A Hettmann T Ossipov D. Monoclonal Antibody Blockade of the Human Eag1 Potassium Channel Function Exerts Antitumor Activity. Cancer Res (2007) 67(15):7343–9. doi: 10.1158/0008-5472.Can-07-0107 17671204

[65] Jahchan NS Dudley JT Mazur PK Flores N Yang D Palmerton A. A Drug Repositioning Approach Identifies Tricyclic Antidepressants as Inhibitors of Small Cell Lung Cancer and Other Neuroendocrine Tumors. Cancer Discovery (2013) 3(12):1364–77. doi: 10.1158/2159-8290.Cd-13-0183 PMC3864571 24078773

[66] Crociani O Guasti L Balzi M Becchetti A Wanke E Olivotto M. Cell Cycle-Dependent Expression of HERG1 and HERG1B Isoforms in Tumor Cells. J Biol Chem (2003) 278(5):2947–55. doi: 10.1074/jbc.M210789200 12431979

[67] Guasti L Crociani O Redaelli E Pillozzi S Polvani S Masselli M. Identification of a Posttranslational Mechanism for the Regulation of Herg1 K+ Channel Expression and Herg1 Current Density in Tumor Cells. Mol Cell Biol (2008) 28(16):5043–60. doi: 10.1128/mcb.00304-08 PMC2519704 18559421

[68] Weaver AK Bomben VC Sontheimer H. Expression and Function of Calcium-Activated Potassium Channels in Human Glioma Cells. Glia (2006) 54(3):223–33. doi: 10.1002/glia.20364 PMC2562223 16817201

[69] Cuddapah VA Sontheimer H. Ion Channels and Transporters [Corrected] in Cancer. 2. Ion Channels and the Control of Cancer Cell Migration. Am J Physiol Cell Physiol (2011) 301(3):C541–9. doi: 10.1152/ajpcell.00102.2011 PMC3174565 21543740

[70] Weber GF. Metabolism in Cancer Metastasis. Int J Cancer (2016) 138(9):2061–6. doi: 10.1002/ijc.29839 26355498

[71] Weber GF. Time and Circumstances: Cancer Cell Metabolism at Various Stages of Disease Progression. Front Oncol (2016) 6:257. doi: 10.3389/fonc.2016.00257 28018856 PMC5149521

[72] Luanpitpong S Talbott SJ Rojanasakul Y Nimmannit U Pongrakhananon V Wang L. Regulation of Lung Cancer Cell Migration and Invasion by Reactive Oxygen Species and Caveolin-1. J Biol Chem (2010) 285(50):38832–40. doi: 10.1074/jbc.M110.124958 PMC2998081 20923773

[73] Hu L Hittelman W Lu T Ji P Arlinghaus R Shmulevich I. NGAL Decreases E-Cadherin-Mediated Cell-Cell Adhesion and Increases Cell Motility and Invasion Through Rac1 in Colon Carcinoma Cells. Lab invest; J Tech Methods Pathol (2009) 89(5):531–48. doi: 10.1038/labinvest.2009.17 PMC7770608 19308044

[74] Mehta KJ Coombes JD Briones-Orta M Manka PP Williams R Patel VB. Iron Enhances Hepatic Fibrogenesis and Activates Transforming Growth Factor-β Signaling in Murine Hepatic Stellate Cells. Am J Med Sci (2018) 355(2):183–90. doi: 10.1016/j.amjms.2017.08.012 29406047

[75] Brookes MJ Boult J Roberts K Cooper BT Hotchin NA Matthews G. A Role for Iron in Wnt Signalling. Oncogene (2008) 27(7):966–75. doi: 10.1038/sj.onc.1210711 17700530

[76] Brown RAM Richardson KL Kabir TD Trinder D Ganss R Leedman PJ. Altered Iron Metabolism and Impact in Cancer Biology, Metastasis, and Immunology. Front Oncol (2020) 10:476. doi: 10.3389/fonc.2020.00476 32328462 PMC7160331

[77] Chi Y Remsik J Kiseliovas V Derderian C Sener U Alghader M. Cancer Cells Deploy Lipocalin-2 to Collect Limiting Iron in Leptomeningeal Metastasis. Sci (New York NY) (2020) 369(6501):276–82. doi: 10.1126/science.aaz2193 PMC7816199 32675368

[78] Shi H Gu Y Yang J Xu L Mi W Yu W. Lipocalin 2 Promotes Lung Metastasis of Murine Breast Cancer Cells. J Exp Clin Cancer Res CR (2008) 27(1):83. doi: 10.1186/1756-9966-27-83 19077278 PMC2614970

[79] Hanai J Mammoto T Seth P Mori K Karumanchi SA Barasch J. Lipocalin 2 Diminishes Invasiveness and Metastasis of Ras-Transformed Cells. J Biol Chem (2005) 280(14):13641–7. doi: 10.1074/jbc.M413047200 15691834

[80] Zhao B Li R Cheng G Li Z Zhang Z Li J. Role of Hepcidin and Iron Metabolism in the Onset of Prostate Cancer. Oncol Lett (2018) 15(6):9953–8. doi: 10.3892/ol.2018.8544 PMC5958801 29844842

[81] Torti SV Torti FM. Ironing Out Cancer. Cancer Res (2011) 71(5):1511–4. doi: 10.1158/0008-5472.Can-10-3614 PMC3079335 21363917

[82] Kim DH Kim JH Kim EH Na HK Cha YN Chung JH. 15-Deoxy-Delta12,14-Prostaglandin J2 Upregulates the Expression of Heme Oxygenase-1 and Subsequently Matrix Metalloproteinase-1 in Human Breast Cancer Cells: Possible Roles of Iron and ROS. Carcinogenesis (2009) 30(4):645–54. doi: 10.1093/carcin/bgp012 19136476

[83] Li Q Liu Q Cheng W Wei H Jiang W Fang E. Heme Oxygenase-1 Inhibits Tumor Metastasis Mediated by Notch1 Pathway in Murine Mammary Carcinoma. Oncol Res (2019) 27(6):643–51. doi: 10.3727/096504018x15415906335771 PMC7848234 30764900

[84] Lin HH Chiang MT Chang PC Chau LY. Myeloid Heme Oxygenase-1 Promotes Metastatic Tumor Colonization in Mice. Cancer Sci (2015) 106(3):299–306. doi: 10.1111/cas.12604 25580731 PMC4376439

[85] Liu Q Wang B Yin Y Chen G Wang W Gao X. Overexpressions of HO-1/HO-1G143H in C57/B6J Mice Affect Melanoma B16F10 Lung Metastases Rather Than Change the Survival Rate of Mice-Bearing Tumours. Exp Biol Med (Maywood NJ) (2013) 238(6):696–704. doi: 10.1177/1535370213490628 23918881

[86] Dolma S Lessnick SL Hahn WC Stockwell BR. Identification of Genotype-Selective Antitumor Agents Using Synthetic Lethal Chemical Screening in Engineered Human Tumor Cells. Cancer Cell (2003) 3(3):285–96. doi: 10.1016/s1535-6108(03)00050-3 12676586

[87] Dixon SJ Lemberg KM Lamprecht MR Skouta R Zaitsev EM Gleason CE. Ferroptosis: An Iron-Dependent Form of Nonapoptotic Cell Death. Cell (2012) 149(5):1060–72. doi: 10.1016/j.cell.2012.03.042 PMC3367386 22632970

[88] Chen Z Zhang D Yue F Zheng M Kovacevic Z Richardson DR. The Iron Chelators Dp44mT and DFO Inhibit TGF-β-Induced Epithelial-Mesenchymal Transition *via* Up-Regulation of N-Myc Downstream-Regulated Gene 1 (NDRG1). J Biol Chem (2012) 287(21):17016–28. doi: 10.1074/jbc.M112.350470 PMC3366822 22453918

[89] Wang J Yin D Xie C Zheng T Liang Y Hong X. The Iron Chelator Dp44mT Inhibits Hepatocellular Carcinoma Metastasis *via* N-Myc Downstream-Regulated Gene 2 (NDRG2)/gp130/STAT3 Pathway. Oncotarget (2014) 5(18):8478–91. doi: 10.18632/oncotarget.2328 PMC4226698 25261367

[90] Menezes SV Fouani L Huang MLH Geleta B Maleki S Richardson A. The Metastasis Suppressor, NDRG1, Attenuates Oncogenic TGF-β and NF-κb Signaling to Enhance Membrane E-Cadherin Expression in Pancreatic Cancer Cells. Carcinogenesis (2019) 40(6):805–18. doi: 10.1093/carcin/bgy178 30561520

[91] Nishitani S Noma K Ohara T Tomono Y Watanabe S Tazawa H. Iron Depletion-Induced Downregulation of N-Cadherin Expression Inhibits Invasive Malignant Phenotypes in Human Esophageal Cancer. Int J Oncol (2016) 49(4):1351–9. doi: 10.3892/ijo.2016.3640 27499208

[92] Li S Zhang J Yang H Wu C Dang X Liu Y. Copper Depletion Inhibits CoCl2-Induced Aggressive Phenotype of MCF-7 Cells *via* Downregulation of HIF-1 and Inhibition of Snail/Twist-Mediated Epithelial-Mesenchymal Transition. Sci Rep (2015) 5:12410. doi: 10.1038/srep12410 26174737 PMC4502431

[93] Parke A Bhattacherjee P Palmer RM Lazarus NR. Characterization and Quantification of Copper Sulfate-Induced Vascularization of the Rabbit Cornea. Am J Pathol (1988) 130(1):173–8. PMC1880543 2447782

[94] Finney L Vogt S Fukai T Glesne D. Copper and Angiogenesis: Unravelling a Relationship Key to Cancer Progression. Clin Exp Pharmacol Physiol (2009) 36(1):88–94. doi: 10.1111/j.1440-1681.2008.04969.x 18505439 PMC4230479

[95] Brem SS Zagzag D Tsanaclis AM Gately S Elkouby MP Brien SE. Inhibition of Angiogenesis and Tumor Growth in the Brain. Suppression of Endothelial Cell Turnover by Penicillamine and the Depletion of Copper, an Angiogenic Cofactor. Am J Pathol (1990) 137(5):1121–42. PMC1877678 1700617

[96] Blockhuys S Zhang X Wittung-Stafshede P. Single-Cell Tracking Demonstrates Copper Chaperone Atox1 to be Required for Breast Cancer Cell Migration. Proc Natl Acad Sci USA (2020) 117(4):2014–9. doi: 10.1073/pnas.1910722117 PMC6995000 31932435

[97] Shanbhag V Jasmer-McDonald K Zhu S Martin AL Gudekar N Khan A. ATP7A Delivers Copper to the Lysyl Oxidase Family of Enzymes and Promotes Tumorigenesis and Metastasis. Proc Natl Acad Sci USA (2019) 116(14):6836–41. doi: 10.1073/pnas.1817473116 PMC6452744 30890638

[98] Erler JT Bennewith KL Cox TR Lang G Bird D Koong A. Hypoxia-Induced Lysyl Oxidase Is a Critical Mediator of Bone Marrow Cell Recruitment to Form the Premetastatic Niche. Cancer Cell (2009) 15(1):35–44. doi: 10.1016/j.ccr.2008.11.012 19111879 PMC3050620

[99] MacDonald G Nalvarte I Smirnova T Vecchi M Aceto N Dolemeyer A. Memo Is a Copper-Dependent Redox Protein With an Essential Role in Migration and Metastasis. Sci Signaling (2014) 7(329):ra56. doi: 10.1126/scisignal.2004870 24917593

[100] Wang HP Wang X Gong LF Chen WJ Hao Z Feng SW. Nox1 Promotes Colon Cancer Cell Metastasis *via* Activation of the ADAM17 Pathway. Eur Rev Med Pharmacol Sci (2016) 20(21):4474–81. 27874952

[101] Yoshii J Yoshiji H Kuriyama S Ikenaka Y Noguchi R Okuda H. The Copper-Chelating Agent, Trientine, Suppresses Tumor Development and Angiogenesis in the Murine Hepatocellular Carcinoma Cells. Int J Cancer (2001) 94(6):768–73. doi: 10.1002/ijc.1537 11745476

[102] Fan C Zhao J Zhao B Zhang S Miao J. Novel Complex of Copper and a Salicylaldehyde Pyrazole Hydrazone Derivative Induces Apoptosis Through Up-Regulating Integrin Beta 4 in Vascular Endothelial Cells. Chem Res Toxicol (2009) 22(9):1517–25. doi: 10.1021/tx900111y 19621939

[103] Lopez J Ramchandani D Vahdat L. Copper Depletion as a Therapeutic Strategy in Cancer. Metal Ions Life Sci (2019) 19. doi: 10.1515/9783110527872-018 30855113

[104] Chan N Willis A Kornhauser N Ward MM Lee SB Nackos E. Influencing the Tumor Microenvironment: A Phase II Study of Copper Depletion Using Tetrathiomolybdate in Patients With Breast Cancer at High Risk for Recurrence and in Preclinical Models of Lung Metastases. Clin Cancer Res an Off J Am Assoc Cancer Res (2017) 23(3):666–76. doi: 10.1158/1078-0432.Ccr-16-1326 27769988

[105] Redman BG Esper P Pan Q Dunn RL Hussain HK Chenevert T. Phase II Trial of Tetrathiomolybdate in Patients With Advanced Kidney Cancer. Clin Cancer Res an Off J Am Assoc Cancer Res (2003) 9(5):1666–72. 12738719

[106] Murray MJ Erickson KL Fisher GL. Effects of Supplemental Zinc on Melanoma Metastasis in Mice. Cancer Lett (1983) 18(3):339–47. doi: 10.1016/0304-3835(83)90245-8 6850565

[107] Liu M Zhang Y Yang J Zhan H Zhou Z Jiang Y. Zinc-Dependent Regulation of ZEB1 and YAP1 Coactivation Promotes Epithelial-Mesenchymal Transition Plasticity and Metastasis in Pancreatic Cancer. Gastroenterology (2021) 160(5):1771–83.e1. doi: 10.1053/j.gastro.2020.12.077 PMC8035249 33421513

[108] Zhang R Zhao G Shi H Zhao X Wang B Dong P. Zinc Regulates Primary Ovarian Tumor Growth and Metastasis Through the Epithelial to Mesenchymal Transition. Free Radical Biol Med (2020) 160:775–83. doi: 10.1016/j.freeradbiomed.2020.09.010 PMC7704937 32927017

[109] Kagara N Tanaka N Noguchi S Hirano T. Zinc and its Transporter ZIP10 are Involved in Invasive Behavior of Breast Cancer Cells. Cancer Sci (2007) 98(5):692–7. doi: 10.1111/j.1349-7006.2007.00446.x PMC11159674 17359283

[110] Manning DL Robertson JF Ellis IO Elston CW McClelland RA Gee JM. Oestrogen-Regulated Genes in Breast Cancer: Association of Pliv1 With Lymph Node Involvement. Eur J Cancer (Oxford Engl 1990) (1994) 30a(5):675–8. doi: 10.1016/0959-8049(94)90543-6 8080686

[111] Kasper G Weiser AA Rump A Sparbier K Dahl E Hartmann A. Expression Levels of the Putative Zinc Transporter LIV-1 Are Associated With a Better Outcome of Breast Cancer Patients. Int J Cancer (2005) 117(6):961–73. doi: 10.1002/ijc.21235 15986450

[112] Li D Stovall DB Wang W Sui G. Advances of Zinc Signaling Studies in Prostate Cancer. Int J Mol Sci (2020) 21(2):667. doi: 10.3390/ijms21020667 PMC7014440 31963946

[113] Franklin RB Feng P Milon B Desouki MM Singh KK Kajdacsy-Balla A. Hzip1 Zinc Uptake Transporter Down Regulation and Zinc Depletion in Prostate Cancer. Mol Cancer (2005) 4:32. doi: 10.1186/1476-4598-4-32 16153295 PMC1243239

[114] Kim YR Kim IJ Kang TW Choi C Kim KK Kim MS. HOXB13 Downregulates Intracellular Zinc and Increases NF-κb Signaling to Promote Prostate Cancer Metastasis. Oncogene (2014) 33(37):4558–67. doi: 10.1038/onc.2013.404 24096478

[115] Guzel RM Ogmen K Ilieva KM Fraser SP Djamgoz MBA. Colorectal Cancer Invasiveness *In Vitro*: Predominant Contribution of Neonatal Nav1.5 Under Normoxia and Hypoxia. J Cell Physiol (2019) 234(5):6582–93. doi: 10.1002/jcp.27399 30341901

[116] Djamgoz MB Onkal R. Persistent Current Blockers of Voltage-Gated Sodium Channels: A Clinical Opportunity for Controlling Metastatic Disease. Recent Patents Anti-Cancer Drug Discov (2013) 8(1):66–84. doi: 10.2174/15748928130107 23116083

[117] Besson P Driffort V Bon É Gradek F Chevalier S Roger S. How do Voltage-Gated Sodium Channels Enhance Migration and Invasiveness in Cancer Cells? Biochim Biophys Acta (2015) 1848(10 Pt B):2493–501. doi: 10.1016/j.bbamem.2015.04.013 25922224

[118] Wang J Lu Z Wu C Li Y Kong Y Zhou R. Evaluation of the Anticancer and Anti-Metastasis Effects of Novel Synthetic Sodium Channel Blockers in Prostate Cancer Cells *In Vitro* and *In Vivo*. Prostate (2019) 79(1):62–72. doi: 10.1002/pros.23711 30242862

[119] Fraser SP Diss JK Chioni AM Mycielska ME Pan H Yamaci RF. Voltage-Gated Sodium Channel Expression and Potentiation of Human Breast Cancer Metastasis. Clin Cancer Res an Off J Am Assoc Cancer Res (2005) 11(15):5381–9. doi: 10.1158/1078-0432.Ccr-05-0327 16061851

[120] Brackenbury WJ Chioni AM Diss JK Djamgoz MB. The Neonatal Splice Variant of Nav1.5 Potentiates *In Vitro* Invasive Behaviour of MDA-MB-231 Human Breast Cancer Cells. Breast Cancer Res Treat (2007) 101(2):149–60. doi: 10.1007/s10549-006-9281-1 PMC4122814 16838113

[121] Nelson M Yang M Millican-Slater R Brackenbury WJ. Nav1.5 Regulates Breast Tumor Growth and Metastatic Dissemination *In Vivo*. Oncotarget (2015) 6(32):32914–29. doi: 10.18632/oncotarget.5441 PMC4741739 26452220

[122] Fraser SP Hemsley F Djamgoz MBA. Caffeic Acid Phenethyl Ester: Inhibition of Metastatic Cell Behaviours *via* Voltage-Gated Sodium Channel in Human Breast Cancer *In Vitro*. Int J Biochem Cell Biol (2016) 71:111–8. doi: 10.1016/j.biocel.2015.12.012 26724521

[123] Liu C Yu M Li Y Wang H Xu C Zhang X. Lidocaine Inhibits the Metastatic Potential of Ovarian Cancer by Blocking Na(V) 1.5-Mediated EMT and FAK/Paxillin Signaling Pathway. Cancer Med (2021) 10(1):337–49. doi: 10.1002/cam4.3621 PMC7826465 33280262

[124] Liu J Liu D Liu JJ Zhao C Yao S Hong L. Blocking the Nav1.5 Channel Using Eicosapentaenoic Acid Reduces Migration and Proliferation of Ovarian Cancer Cells. Int J Oncol (2018) 53(2):855–65. doi: 10.3892/ijo.2018.4437 29901108

[125] Yildirim S Altun S Gumushan H Patel A Djamgoz MBA. Voltage-Gated Sodium Channel Activity Promotes Prostate Cancer Metastasis *In Vivo*. Cancer Lett (2012) 323(1):58–61. doi: 10.1016/j.canlet.2012.03.036 22484465

[126] Nakajima T Kubota N Tsutsumi T Oguri A Imuta H Jo T. Eicosapentaenoic Acid Inhibits Voltage-Gated Sodium Channels and Invasiveness in Prostate Cancer Cells. Br J Pharmacol (2009) 156(3):420–31. doi: 10.1111/j.1476-5381.2008.00059.x PMC2697676 19154441

[127] Baptista-Hon DT Robertson FM Robertson GB Owen SJ Rogers GW Lydon EL. Potent Inhibition by Ropivacaine of Metastatic Colon Cancer SW620 Cell Invasion and NaV1.5 Channel Function. Br J Anaesthesia (2014) 113 Suppl 1:i39–48. doi: 10.1093/bja/aeu104 24852501

[128] Campbell TM Main MJ Fitzgerald EM. Functional Expression of the Voltage-Gated Na⁺-Channel Nav1.7 Is Necessary for EGF-Mediated Invasion in Human Non-Small Cell Lung Cancer Cells. J Cell Sci (2013) 126(Pt 21):4939–49. doi: 10.1242/jcs.130013 23986482

[129] Luo Q Wu T Wu W Chen G Luo X Jiang L. The Functional Role of Voltage-Gated Sodium Channel Nav1.5 in Metastatic Breast Cancer. Front Pharmacol (2020) 11:1111. doi: 10.3389/fphar.2020.01111 32792949 PMC7393602

[130] Chen B Zhang C Wang Z Chen Y Xie H Li S. Mechanistic Insights Into Nav1.7-Dependent Regulation of Rat Prostate Cancer Cell Invasiveness Revealed by Toxin Probes and Proteomic Analysis. FEBS J (2019) 286(13):2549–61. doi: 10.1111/febs.14823 30927332

[131] Angus M Ruben P. Voltage Gated Sodium Channels in Cancer and Their Potential Mechanisms of Action. Channels (Austin Tex) (2019) 13(1):400–9. doi: 10.1080/19336950.2019.1666455 PMC6768049 31510893

[132] Djamgoz MBA Fraser SP Brackenbury WJ. *In Vivo* Evidence for Voltage-Gated Sodium Channel Expression in Carcinomas and Potentiation of Metastasis. Cancers (2019) 11(11):1675. doi: 10.3390/cancers11111675 PMC6895836 31661908

[133] Chioni AM Brackenbury WJ Calhoun JD Isom LL Djamgoz MB. A Novel Adhesion Molecule in Human Breast Cancer Cells: Voltage-Gated Na+ Channel Beta1 Subunit. Int J Biochem Cell Biol (2009) 41(5):1216–27. doi: 10.1016/j.biocel.2008.11.001 PMC2678854 19041953

[134] Brackenbury WJ. Voltage-Gated Sodium Channels and Metastatic Disease. Channels (Austin Tex) (2012) 6(5):352–61. doi: 10.4161/chan.21910 PMC3508774 22992466

[135] Lee A Fraser SP Djamgoz MBA. Propranolol Inhibits Neonatal Nav1.5 Activity and Invasiveness of MDA-MB-231 Breast Cancer Cells: Effects of Combination With Ranolazine. J Cell Physiol (2019) 234(12):23066–81. doi: 10.1002/jcp.28868 31222761

[136] Friedl P Alexander S. Cancer Invasion and the Microenvironment: Plasticity and Reciprocity. Cell (2011) 147(5):992–1009. doi: 10.1016/j.cell.2011.11.016 22118458

[137] Gillet L Roger S Besson P Lecaille F Gore J Bougnoux P. Voltage-Gated Sodium Channel Activity Promotes Cysteine Cathepsin-Dependent Invasiveness and Colony Growth of Human Cancer Cells. J Biol Chem (2009) 284(13):8680–91. doi: 10.1074/jbc.M806891200 PMC2659227 19176528

[138] Mao W Zhang J Körner H Jiang Y Ying S. The Emerging Role of Voltage-Gated Sodium Channels in Tumor Biology. Front Oncol (2019) 9:124. doi: 10.3389/fonc.2019.00124 30895169 PMC6414428

[139] Carrithers MD Chatterjee G Carrithers LM Offoha R Iheagwara U Rahner C. Regulation of Podosome Formation in Macrophages by a Splice Variant of the Sodium Channel SCN8A. J Biol Chem (2009) 284(12):8114–26. doi: 10.1074/jbc.M801892200 PMC2658105 19136557

[140] Koltai T. Voltage-Gated Sodium Channel as a Target for Metastatic Risk Reduction With Re-Purposed Drugs. F1000Research (2015) 4:297. doi: 10.12688/f1000research.6789.1 27408684 PMC4920216

[141] House CD Vaske CJ Schwartz AM Obias V Frank B Luu T. Voltage-Gated Na+ Channel SCN5A Is a Key Regulator of a Gene Transcriptional Network That Controls Colon Cancer Invasion. Cancer Res (2010) 70(17):6957–67. doi: 10.1158/0008-5472.Can-10-1169 PMC2936697 20651255

[142] Driffort V Gillet L Bon E Marionneau-Lambot S Oullier T Joulin V. Ranolazine Inhibits NaV1.5-Mediated Breast Cancer Cell Invasiveness and Lung Colonization. Mol Cancer (2014) 13:264. doi: 10.1186/1476-4598-13-264 25496128 PMC4295566

[143] Gumushan Aktas H Akgun T. Naringenin Inhibits Prostate Cancer Metastasis by Blocking Voltage-Gated Sodium Channels. Biomed Pharmacother = Biomed Pharmacother (2018) 106:770–5. doi: 10.1016/j.biopha.2018.07.008 29990870

[144] Yang M Kozminski DJ Wold LA Modak R Calhoun JD Isom LL. Therapeutic Potential for Phenytoin: Targeting Na(v)1.5 Sodium Channels to Reduce Migration and Invasion in Metastatic Breast Cancer. Breast Cancer Res Treat (2012) 134(2):603–15. doi: 10.1007/s10549-012-2102-9 PMC3401508 22678159

[145] Nelson M Yang M Dowle AA Thomas JR Brackenbury WJ. The Sodium Channel-Blocking Antiepileptic Drug Phenytoin Inhibits Breast Tumour Growth and Metastasis. Mol Cancer (2015) 14(1):13. doi: 10.1186/s12943-014-0277-x 25623198 PMC4320839

[146] Litan A Langhans SA. Cancer as a Channelopathy: Ion Channels and Pumps in Tumor Development and Progression. Front Cell Neurosci (2015) 9:86. doi: 10.3389/fncel.2015.00086 25852478 PMC4362317

[147] Ransom CB O'Neal JT Sontheimer H. Volume-Activated Chloride Currents Contribute to the Resting Conductance and Invasive Migration of Human Glioma Cells. J Neurosci Off J Soc Neurosci (2001) 21(19):7674–83. doi: 10.1523/jneurosci.21-19-07674.2001 PMC6762888 11567057

[148] Haas BR Sontheimer H. Inhibition of the Sodium-Potassium-Chloride Cotransporter Isoform-1 Reduces Glioma Invasion. Cancer Res (2010) 70(13):5597–606. doi: 10.1158/0008-5472.Can-09-4666 PMC2896443 20570904

[149] Zhou Y Sun W Chen N Xu C Wang X Dong K. Discovery of NKCC1 as a Potential Therapeutic Target to Inhibit Hepatocellular Carcinoma Cell Growth and Metastasis. Oncotarget (2017) 8(39):66328–42. doi: 10.18632/oncotarget.20240 PMC5630415 29029515

[150] Sontheimer H. An Unexpected Role for Ion Channels in Brain Tumor Metastasis. Exp Biol Med (Maywood NJ) (2008) 233(7):779–91. doi: 10.3181/0711-mr-308 PMC2557067 18445774

[151] Papadopoulos MC Saadoun S Verkman AS. Aquaporins and Cell Migration. Pflugers Archiv Eur J Physiol (2008) 456(4):693–700. doi: 10.1007/s00424-007-0357-5 17968585 PMC3595095

[152] Stock C Schwab A. Ion Channels and Transporters in Metastasis. Biochim Biophys Acta (2015) 1848(10 Pt B):2638–46. doi: 10.1016/j.bbamem.2014.11.012 25445667

[153] Johnson MD O'Connell M. Na-K-2Cl Cotransporter and Aquaporin 1 in Arachnoid Granulations, Meningiomas, and Meningiomas Invading Dura. Hum Pathol (2013) 44(6):1118–24. doi: 10.1016/j.humpath.2012.09.020 23317544

