# Supplementary material for: Alterations of Ion Homeostasis in Cancer Metastasis: Implications for Treatment
Source: Front Oncol. 2021 Dec 20;11:765329. doi: 10.3389/fonc.2021.765329 (PMC8721045; doi:10.3389/fonc.2021.765329)
Supplement: Supplementary file 1 [file DataSheet_1.pdf]

Table 1: Commonly deregulated genes in metastases related to ion homeostasis. Select genes are up- or down- regulated compared to the primary tumor or the host tissue in all or most of the target sites. On the basis of 653 metastasis-associated gene expression profiles in GEO (2), this table displays the genes pertaining to ion homeostasis. Those sets of genes seem to constitute essential mediators of metastasis, possibly conveying the ability to survive and expand in a non-cognate microenvironment. Genes different from the primary tumor may represent necessary contributors for metastasis initiation, while the genes that distinguish metastases from the target host site are likely reflective of metastasis outgrowth. **A)** Genes significantly up-regulated in the metastases over host tissue. **B)** Genes significantly down-regulated in the metastases compared to host tissue. Liver = li, peritoneum = pe, ovary = ov, lymph node = ln, adrenal = ad, bone = bo, lung = lu. **C)** Genes significantly up-regulated in the metastases over primary tumors. **D)** Genes significantly down-regulated in the metastases over primary tumors. Ovary = ov, breast = br, prostate = pr, kidney = ki, stomach = st, colon = co.

Gene symbol	Tissues of gene listed	# Tissues of gene listed	SD of each gene	Na	K	Ca	Mg	Mn	Fe	Zn	Cl	other
MMP26	breast:prostate:kidney:	3	0.03									
S100A8	breast:kidney:	2	0.02									
ADCY7	ovary:breast:kidney:	3	0.10									
KCNMA1	kidney:	1	0.00									
CLIC4	breast:prostate:	2	0.04									
LPO	breast:prostate:kidney:	3	0.06									
ANXA1	breast:kidney:	2	0.01									
ANXA3	breast:	1	0.00									
CCL19	kidney:	1	0.00									
CPNE3	breast:prostate:kidney:	3	0.05									
DCHS1	breast:prostate:kidney:	3	0.06									
DNAH7	ovary:breast:prostate:	3	0.15									
HRH4	breast:prostate:kidney:	3	0.03									
HSPA5	ovary:prostate:	2	0.02									
TBRG4	breast:kidney:	2	0.01									
TRPC6	breast:kidney:	2	0.02									
TRPC7	ovary:breast:prostate:kidney:	4	0.11									
ALAD	breast:	1	0.00									lead, cobalt, copper, aluminum
BBOX1	breast:prostate:kidney:	3	0.00									
POLD1	breast:prostate:kidney:	3	0.14									
KCND3	breast:prostate:kidney:	3	0.03									
KCNK3	breast:	1	0.00									
CPS1	breast:prostate:kidney:	3	0.10									
CRIP1	breast:prostate:	2	0.00									
NEIL3	breast:prostate:kidney:	3	0.03									
SLC39A2	breast:prostate:kidney:	3	0.04									
TRIM28	breast:prostate:kidney:	3	0.02									
ZBTB24	ovary:breast:prostate:	3	0.09									
ZFAND3	breast:prostate:kidney:	3	0.00									
ZFP36L1	breast:prostate:kidney:	3	0.09									
ZNF34	breast:prostate:kidney:	3	0.04									
ZNF667	breast:prostate:kidney:	3	0.06									
CNNM2	breast:prostate:kidney:	3	0.06									cobalt
ENO2	breast:prostate:kidney:	3	0.03									
EYA3	breast:prostate:kidney:	3	0.10									
LIG1	ovary:breast:kidney:	3	0.04									
MAPK6	breast:prostate:kidney:	3	0.02									
C1QA	breast:prostate:kidney:	3	0.09									
TDO2	breast:prostate:kidney:	3	0.02									
CYP1A1	prostate:	1	0.00									copper
DTYMK	breast:kidney:	2	0.02									cadmium
IGFBP2	breast:kidney:	2	0.00									lithium
KCNC2	breast:kidney:	2	0.05									amine, nitric oxide
PTK2B	breast:kidney:	2	0.03									nitric oxide
LOXL2	prostate:kidney:	2	0.00									copper
SCLY	breast:prostate:kidney:	3	0.05									selenium

Gene symbol	Tissues of gene listed	# Tissues of gene listed	SD of each gene	Na	K	Ca	Mg	Mn	Fe	Zn	Cl	other
MNAT1	kidney:	1	0.00									
S100A7	ovary:prostate:kidney:	3	0.02									
KCNMA1	breast:	1	0.00									
ANXA10	breast:	1	0.00									
ANXA2	prostate:	1	0.00									
ANXA5	breast:prostate:	2	0.08									
CACNG3	breast:prostate:kidney:	3	0.05									
CALCR	breast:prostate:kidney:	3	0.03									
CAPN7	breast:prostate:kidney:	3	0.02									
CCL19	breast:	1	0.00									
CCL8	breast:prostate:kidney:	3	0.05									
F9	breast:prostate:kidney:	3	0.04									
GPR98	breast:prostate:	2	0.05									
GSN	breast:prostate:	2	0.06									
ITGA5	breast:prostate:	2	0.07									
MLC1	breast:prostate:kidney:	3	0.04									
SNTA1	breast:kidney:	2	0.03									
TGM2	breast:prostate:kidney:	3	0.07									
TNNT3	breast:	1	0.00									
TRPV2	breast:prostate:kidney:	3	0.10									
EGR4	breast:prostate:kidney:	3	0.02									
LMO1	breast:prostate:kidney:	3	0.03									
NAALADL1	breast:prostate:kidney:	3	0.03									
SEC24C	breast:prostate:kidney:	3	0.05									
SF3A2	breast:prostate:kidney:	3	0.29									
TNFSF10	breast:prostate:kidney:	3	0.03									
YAF2	breast:prostate:	2	0.03									
ZBTB6	breast:prostate:kidney:	3	0.05									
ATP7B	breast:	1	0.00									copper
ERCC5	breast:prostate:kidney:	3	0.02									
TERT	breast:prostate:	2	0.13									
GCLC	breast:	1	0.00									cadmium
GPHN	breast:kidney:	2	0.07									nitrate
B3GALT2	breast:prostate:kidney:	3	0.04									
EIF2S1	breast:	1	0.00									
PPP2CA	breast:	1	0.00									
ATP1B3	breast:prostate:kidney:	3	0.03									
CD163	breast:prostate:kidney:	3	0.04									
CYP1A1	breast:	1	0.00									
GLRA3	breast:	1	0.00									
PTCH1	breast:prostate:	2	0.06									
SLC12A1	breast:kidney:	2	0.21									
SLC12A2	breast:	1	0.00									
NEDD4L	breast:prostate:	2	0.11									
NEDD4	breast:	1	0.00									
ATF1	breast:prostate:	2	0.02									cobalt
PPARGC1A	prostate:kidney:	2	0.01									nitrite
TCN2	breast:prostate:kidney:	3	0.05									cobalt
TNFSF4	breast:prostate:	2	0.09									nitrogen dioxide

Gene symbol	Tissues of gene listed	# Tissues of gene listed	SD of each gene	Na	K	Ca	Mg	Mn	Fe	Zn	Cl	other
MKNK2	liver:peritoneum:ovary:bone:adrenal:	5	0.10									
MYO9B	liver:peritoneum:ovary:lymph node:bone:adrenal:	6	0.06									
P2RX1	peritoneum:liver:ovary:adrenal:lung:	5	0.06									
PGS1	peritoneum:liver:ovary:bone:adrenal:	5	0.09									
TRPC1	peritoneum:lymph node:adrenal:	3	0.19									
NEDD4	peritoneum:bone:adrenal:	3	0.08									
A2M	peritoneum:liver:ovary:bone:adrenal:	5	0.18									
ANXA11	peritoneum:bone:adrenal:	3	0.04									
CACNG3	peritoneum:ovary:lymph node:adrenal:lung:	5	0.11									
CCL19	liver:bone:adrenal:	3	0.23									
JPH2	liver:peritoneum:ovary:bone:adrenal:	5	0.08									
P2RX7	peritoneum:liver:adrenal:	3	0.10									
PLA2G2A	liver:peritoneum:ovary:bone:adrenal:	5	0.09									
PLCD1	liver:peritoneum:ovary:bone:adrenal:	5	0.11									
PPIF	liver:ovary:peritoneum:adrenal:	4	0.27									
SF3B3	liver:peritoneum:adrenal:lung:	4	0.13									
TBRG4	liver:bone:adrenal:	3	0.09									
TNNC1	bone:adrenal:lung:	3	0.16									
TNNT1	ovary:lymph node:lung:	3	0.06									
TNNT3	peritoneum:liver:adrenal:	3	0.04									
GSN	peritoneum:bone:adrenal:	3	0.11									cadmium
ADA	liver:peritoneum:ovary:bone:adrenal:	5	0.12									
BSN	liver:peritoneum:ovary:lymph node:bone:adrenal:lung:	7	0.06									
CHN2	liver:peritoneum:lymph node:adrenal:lung:	5	0.11									
PPP1R10	peritoneum:ovary:bone:adrenal:lung:	5	0.09									
PRDM14	peritoneum:ovary:liver:adrenal:lung:	5	0.07									
RNF10	liver:peritoneum:ovary:bone:adrenal:	5	0.09									
RREB1	liver:peritoneum:bone:adrenal:lung:	5	0.05									
SALL1	peritoneum:liver:ovary:adrenal:lung:	5	0.10									
SIRT4	peritoneum:liver:ovary:lymph node:adrenal:lung:	6	0.07									
SLC30A4	liver:ovary:peritoneum:adrenal:	4	0.13									
SLC30A6	peritoneum:liver:adrenal:	3	0.06									
SP110	peritoneum:liver:lymph node:bone:adrenal:lung:	6	0.08									
TNKS	peritoneum:liver:ovary:bone:adrenal:	5	0.03									
USP4	peritoneum:liver:lymph node:bone:adrenal:	5	0.19									
ZKSCAN1	liver:ovary:lymph node:bone:adrenal:lung:	6	0.14									
ZNF551	ovary:liver:peritoneum:lymph node:adrenal:lung:	6	0.13									
DAK	liver:peritoneum:ovary:bone:adrenal:	5	0.11									cobalt
TAF1	peritoneum:ovary:lymph node:bone:adrenal:	5	0.12									
TAOK2	liver:peritoneum:bone:adrenal:lung:	5	0.08									
GALNT10	peritoneum:liver:ovary:lymph node:adrenal:lung:	6	0.08									
ABAT	peritoneum:liver:ovary:lymph node:bone:	5	0.07									
G6PD	liver:peritoneum:bone:adrenal:lung:	5	0.08									
GLRA3	peritoneum:ovary:liver:	3	0.08									
SLC12A1	peritoneum:liver:	2	0.00									
SLC12A2	peritoneum:liver:ovary:adrenal:	4	0.07									
NEDD4L	peritoneum:liver:bone:adrenal:	4	0.01									
SLC9A3	liver:peritoneum:bone:adrenal:lung:	5	0.10									
SNTA1	peritoneum:liver:ovary:lymph node:bone:lung:	6	0.08									
HSF1	ovary:peritoneum:bone:	3	0.10									copper, cadmium
ATF3	peritoneum:liver:bone:lung:	4	0.03									arsenic
CYP1A1	peritoneum:adrenal:lung:	3	0.15									iron
EIF2B5	peritoneum:liver:ovary:lymph node:bone:adrenal:	6	0.14									lithium
OGG1	peritoneum:liver:ovary:	3	0.17									cadmium
TNFSF4	liver:ovary:lung:	3	0.09									NO2
YAF2	liver:peritoneum:ovary:lymph node:bone:adrenal:lung:	7	0.06									metal

Gene symbol	Tissues of gene listed	# Tissues of gene listed	SD of each gene	Na	K	Ca	Mg	Mn	Fe	Zn	Cl	other
GDPD3	liver:peritoneum:ovary:lymph node:adrenal:	5	0.14									
ADAMTS1	peritoneum:liver:ovary:lymph node:adrenal:	5	0.05									
S100A8	peritoneum:bone:adrenal:lung:	4	0.07									
TRPM2	liver:peritoneum:adrenal:	3	0.20									
MCOLN1	peritoneum:ovary:bone:adrenal:	4	0.17									
TTYH1	liver:lymph node:bone:adrenal:lung:	5	0.09									
KCNMA1	peritoneum:bone:adrenal:	3	0.18									
TRPC6	liver:peritoneum:adrenal:	3	0.12									
TRPC4	ovary:lymph node:bone:adrenal:	4	0.11									
ANXA13	peritoneum:liver:adrenal:	3	0.17									
ANXA3	liver:peritoneum:adrenal:	3	0.25									
ANXA7	liver:peritoneum:adrenal:	3	0.26									
FSTL4	liver:lymph node:ovary:bone:lung:	5	0.07									
S100A1	ovary:liver:lymph node:bone:adrenal:	5	0.12									
SLC25A13	bone:	1	0.00									
TGM1	liver:peritoneum:ovary:lymph node:bone:lung:	6	0.10									
TNNT1	liver:peritoneum:bone:	3	0.13									
TNNT3	ovary:bone:	2	0.30									
TRPC4AP	peritoneum:ovary:lymph node:bone:adrenal:lung:	6	0.13									
PTK2B	liver:bone:adrenal:	3	0.12									lithium
PRKD2	peritoneum:lymph node:bone:adrenal:lung:	5	0.15									
RFK	liver:peritoneum:ovary:lymph node:adrenal:	5	0.09									
ADH1A	peritoneum:liver:lymph node:ovary:bone:adrenal:	6	0.12									
ATF2	liver:peritoneum:adrenal:	3	0.16									
CHFR	peritoneum:liver:ovary:bone:adrenal:	5	0.05									
KHK	liver:peritoneum:adrenal:	3	0.25									
RPS27L	peritoneum:ovary:lymph node:bone:lung:	5	0.03									
ZFP36L1	liver:ovary:bone:adrenal:lung:	5	0.12									
ZFYVE16	liver:peritoneum:lymph node:adrenal:lung:	5	0.17									
ZFYVE21	peritoneum:ovary:bone:adrenal:lung:	5	0.19									
ZNF35	peritoneum:liver:ovary:lymph node:bone:adrenal:	6	0.08									
ZNF638	liver:ovary:bone:adrenal:lung:	5	0.06									
ZNF667	liver:peritoneum:ovary:lymph node:adrenal:	5	0.11									
ZSCAN16	liver:peritoneum:lymph node:adrenal:lung:	5	0.11									
ALAD	peritoneum:liver:adrenal:	3	0.18									metal ion
SORD	bone:adrenal:lung:	3	0.19									copper, cadmium
PPP2CA	liver:bone:adrenal:	3	0.06									lead
CYP1A1	liver:bone:	2	0.10									
CYP46A1	liver:peritoneum:ovary:lymph node:bone:adrenal:	6	0.19									
SCD5	peritoneum:liver:bone:adrenal:lung:	5	0.15									
SLC25A28	peritoneum:lymph node:bone:adrenal:lung:	5	0.09									
CLIC4	peritoneum:ovary:bone:	3	0.19									
SLC12A1	bone:	1	0.00									
SLC12A2	bone:	1	0.00									
SLC1A1	peritoneum:ovary:liver:lymph node:bone:lung:	6	0.14									
GPHN	peritoneum:liver:adrenal:	3	0.22									metal ion
IGFBP2	peritoneum:liver:adrenal:lung:	4	0.12									lithium
IL1A	liver:peritoneum:adrenal:	3	0.19									copper
LOXL2	liver:peritoneum:adrenal:	3	0.30									copper
TNFSF4	peritoneum:bone:adrenal:	3	0.03									nitrogen dioxide
